# Pharmacologic Treatments for Dementia and the Risk of Developing Age-Related Macular Degeneration

**DOI:** 10.1001/jamanetworkopen.2024.41166

**Published:** 2024-10-24

**Authors:** Jingya Wang, Christina Antza, Wen Hwa Lee, Jesse Coker, Pearse A. Keane, Alastair K. Denniston, Krishnarajah Nirantharakumar, Nicola J. Adderley

**Affiliations:** 1 Department of Applied Health Sciences, University of Birmingham, Birmingham, United Kingdom; 2 Action Against Age-Related Macular Degeneration, London, United Kingdom; 3 NIHR (National Institute for Health and Care Research) Moorfields Biomedical Research Centre, Moorfields Eye Hospital NHS Foundation Trust and Institute of Ophthalmology, University College London, London, United Kingdom; 4 University Hospitals Birmingham NHS Foundation Trust, Birmingham, United Kingdom; 5 Health Data Research UK, London, United Kingdom; 6 NIHR Birmingham Biomedical Research Centre, Birmingham, United Kingdom

## Abstract

**Importance:**

Age-related macular degeneration (AMD) is the leading cause of blindness among people aged 50 years or older worldwide. There is a need for new strategies for the prevention and treatment of AMD. There is some limited evidence to suggest the possibility of a protective association of dementia medications with the development of some types of AMD, but the evidence is weak.

**Objective:**

To investigate whether the dementia medications memantine and donepezil are associated with the risk of developing AMD.

**Design, Setting, and Participants:**

Three population-based cohort studies were performed using data from the Clinical Practice Research Datalink GOLD and Aurum databases from May 15, 2002, to June 21, 2022. Participants included individuals with dementia (vascular dementia, nonvascular dementia, or Alzheimer disease) aged 40 years or older. Statistical analysis was carried out between February and November 2023.

**Exposures:**

Exposures were dementia medications. Cohort 1 compared patients prescribed donepezil with those prescribed rivastigmine or galantamine using the new-user design. Cohort 2 compared memantine with donepezil, rivastigmine, or galantamine using the prevalent new-user design. In a sensitivity analysis, cohort 3 compared memantine with rivastigmine or galantamine only.

**Main Outcomes and Measures:**

New diagnosis of AMD.

**Results:**

There were 132 846 individuals (mean [SD] age, 80.4 [7.6] years; 61.8% women; mean [SD] body mass index [BMI], 25.5 [4.6]) with a diagnosis of dementia included in cohort 1, 159 419 individuals (mean [SD] age, 81.2 [7.6] years; 59.7% women; mean [SD] body mass index [BMI], 25.6 [4.7]) with a diagnosis of dementia included in cohort 2, and 92 328 individuals with a diagnosis of dementia included in cohort 3 (mean [SD] age, 80.9 [7.7] years; 58.5% women; mean [SD] body mass index [BMI], 25.5 [4.7]). The adjusted hazard ratio (HR) for donepezil compared with rivastigmine or galantamine (cohort 1) was 0.95 (95% CI, 0.67-1.35). The adjusted HR for memantine compared with donepezil, rivastigmine, or galantamine (cohort 2) was 1.03 (95% CI, 0.83-1.27). The adjusted HR for memantine vs rivastigmine or galantamine only (cohort 3) was 1.24 (95% CI, 0.83-1.86).

**Conclusions and Relevance:**

This cohort study of patients with dementia found no significant associations between memantine or donepezil compared with other dementia medications and the risk of development of AMD. Further research is recommended to examine any possible pathophysiological protective action of memantine and other dementia medications against the development of AMD.

## Key Points

**Question:**

Are commonly prescribed dementia medications (memantine and donepezil) associated with a reduced risk of developing age-related macular degeneration (AMD)?

**Findings:**

This cohort study using clinical practice data on 384 593 patients from 3 cohorts derived from the Clinical Practice Research Datalink in the UK found no significant association between dementia medications and development of AMD among individuals with dementia.

**Meaning:**

This study suggests that there is no protective (or harmful) association of memantine or donepezil with the risk of developing AMD among patients with dementia.

## Teaser

This cohort study investigates whether dementia medications memantine and donepezil are associated with the risk of developing age-related macular degeneration.

## Introduction

The global population is aging as a result of increasing life expectancy. It is estimated that the world’s population older than 60 years of age will reach 22% of the population by 2050.^1^ Consequently, the prevention and treatment of diseases associated with aging populations is a priority. One such disease is age-related macular degeneration (AMD), which remains the leading cause of blindness among people aged 50 years or older worldwide,^2,3,4^ highlighting the need for new strategies for both prevention and treatment of AMD.

Dementia is a syndrome characterized by a decline in cognitive function due to brain injury, causing disability as a result of both memory and mobility loss.^5^ There is strong evidence supporting an association between dementia and other age-related diseases of the eyes, including AMD, cataracts, glaucoma, and diabetic retinopathy.^6^ This association of age-related diseases of the brain with age-related diseases of the eyes is unsurprising, considering the shared pathophysiological mechanisms, such as microvascular dysfunction and dysregulation of neurovascular coupling, as well as the alterations of the blood-brain and blood-retinal barriers, possibly under the umbrella of “inflamm-aging” (ie, an age-related increase in the levels of proinflammatory markers in blood and tissues) and oxidative stress.^7^

Taking into account the association between AMD and dementia^6^ and their shared pathophysiology mechanisms,^7^ as well as the antioxidant and neuroprotective effects of dementia treatment,^8,9^ the hypothesis that dementia medications could also be associated with AMD seems logical. However, there is currently limited evidence regarding the potential association between dementia medications and the development of AMD. A previous exploratory analysis of case-control studies,^10^ as well as a series of nested case-control studies conducted by a team at Harvard Medical School,^11^ suggested that donepezil and memantine might have a potential protective effect on the progression to neovascular AMD. However, given the case-control design and the exploratory nature of these 2 studies, there is the potential for results to be affected by bias and confounding, and it is important that the associations are further investigated using a more robust study design.

Therefore, the aim of this study was to investigate whether donepezil or memantine, compared with other dementia medications, is associated with a reduced risk of subsequent development of AMD in a cohort of patients with dementia, using a retrospective cohort study in a large nationally representative UK dataset.

## Methods

### Study Design

We performed 3 population-based cohort studies to investigate the association between pharmacologic treatments for dementia and the risk of developing AMD among individuals with dementia. Cohort 1 compared the risk of developing AMD between individuals prescribed donepezil and those prescribed rivastigmine or galantamine (all first-line treatments for dementia) using the new-user design. Cohort 2 compared the risk of developing AMD between individuals prescribed memantine (a second-line treatment for dementia) and those prescribed donepezil, rivastigmine, or galantamine (first-line treatments for dementia) using the prevalent new-user design. There is no other commonly used second-line treatment for dementia that can be used as comparator drug for memantine. In a sensitivity analysis to assess the association of including donepezil in the comparator group (of cohort 2) with developing AMD, cohort 3 compared individuals prescribed memantine with those prescribed rivastigmine or galantamine only. The study period was May 15, 2002 (the date at which all 4 pharmacologic treatments for dementia were approved by the European Medicines Agency), to June 21, 2022, for Clinical Practice Research Datalink (CPRD) GOLD and March 21, 2022, for CPRD Aurum (latest available data at the time of analysis). This study followed the Strengthening the Reporting of Observational Studies in Epidemiology (STROBE) reporting guideline for cohort studies.^12^ Observational research using CPRD data was approved by the National Research Ethics Service Committee. This study was approved by the independent Research Data Governance Committee. Because this study used the CPRD database of pseudonymized patient electronic health care records, patients’ informed consent was not required.

### Data Source

The data for this study were derived from the CPRD GOLD and CPRD Aurum databases, which contain computerized primary care records covering approximately 16.8 million active participants from general practices that use Vision/EMIS Web electronic medical records software across the UK.^13^ Information relating to symptoms, examinations, investigations, and diagnoses are recorded as clinical codes (Read Codes in CPRD GOLD and SNOMED-CT [Systemized Nomenclature of Medicine—Clinical Terms] codes in CPRD Aurum).^14^ All prescriptions issued in primary care are also recorded. To maximize data quality, general practices in CPRD GOLD were eligible 12 months after the up-to-standard date (the up-to-standard date is not currently available for CPRD Aurum, but data are considered reliable for the time period of this study).^15^ To avoid duplication of patients where practices may have changed electronic health records system and therefore moved from one database to another, practices that appeared in both databases were excluded from the GOLD analysis and included only in the Aurum analysis.

### Study Population

Individuals aged 40 years or older with a preexisting diagnosis of dementia (vascular dementia, nonvascular dementia, or Alzheimer disease; eTable 1 in Supplement 1) who had a record of pharmacologic treatments for dementia (donepezil, rivastigmine, galantamine, or memantine; eTable 2 in Supplement 1) were eligible for inclusion in the study. Individuals who developed AMD within 3 months after the first diagnosis date of dementia or those with a record of AMD at baseline were not included in this study. Participants must have been registered with an eligible practice for 12 months prior to inclusion in the study to ensure complete recording of data on exposure, outcome, and covariates.

#### Cohort 1

Individuals prescribed donepezil (exposed group) were compared with those prescribed rivastigmine or galantamine (comparator group). Individuals prescribed both donepezil and rivastigmine or galantamine on the same day were excluded to ensure the exposed and comparator groups were mutually exclusive. The index date was defined as the first prescription date of donepezil, rivastigmine, or galantamine.

#### Cohort 2

Individuals with at least 1 prescription of memantine (exposed group) were compared with those who were prescribed any first-line treatment: donepezil, rivastigmine, or galantamine (unexposed pool). Individuals who initiated first-line treatment and memantine on the same day were excluded from this analysis. The index date of the exposed group was defined as the first prescription date of memantine. For each individual, the number of previous prescriptions of first-line dementia pharmacologic treatments (donepezil, rivastigmine, or galantamine) before the index date (initiation of memantine) was calculated; multiple prescriptions issued on the same day were counted as 1 prescription. Up to 2 individuals in the comparator pool were assigned an index date based on the number of previous prescriptions of first-line dementia pharmacologic treatments (±1) and the calendar year of each corresponding exposed individual and were matched using time-dependent propensity score (PS) matching (eFigure 1 in Supplement 1). Individuals could serve as a comparator prior to being initiated on memantine.

#### Cohort 3

Individuals with at least 1 prescription of memantine (exposed group) were compared with those who were prescribed rivastigmine or galantamine only (unexposed pool). The design of the cohort 3 analysis and the selection of the comparator cohort followed the same method as that for cohort 2, except that individuals prescribed donepezil were not included in the comparator group.

### Outcome

The outcome of interest was a new diagnosis of AMD. Any individual who received a diagnosis of AMD (the outcome) prior to the index date was excluded. Diagnosis of AMD was identified by clinical codes (Read Codes in CPRD GOLD and SNOMED-CT codes in CPRD Aurum; eTable 3 in Supplement 1).

### Follow-Up Period

All participants were followed up from the index date until the earliest date of the following events: diagnosis of AMD, death, individual left practice, practice ceased contributing to the database, 3 years after the last prescription of exposure treatment, the date when individuals switched to treatment in the other group, or study end date (June 21, 2022, for CPRD GOLD; March 21, 2022, for CPRD Aurum). In cohort 2, individuals in the memantine group were allowed to receive comparator treatments and were not censored if they were prescribed a comparator treatment during the study period, as long as they continued to be prescribed memantine.

### Covariates

The latest available covariate data recorded prior to the index date were obtained and used for weighting in the exposure model and for adjustment in the outcome model. Covariates included sociodemographic characteristics (age, sex, and race and ethnicity), behavioral risk factors (body mass index [BMI; calculated as weight in kilograms divided by height in meters squared], smoking status, and drinking status), dementia-related characteristics (duration of disease and type of dementia), comorbidities (hypertension, chronic kidney disease, ischemic heart disease, stroke or transient ischemic attack, myocardial infarction, heart failure, atrial fibrillation, peripheral vascular disease, aortic atheroma or plaque, type 1 or type 2 diabetes, peripheral neuropathy, diabetic retinopathy, diabetic foot disease, osteoporosis, arthritis, gout, hypothyroidism, hyperthyroidism, depression, anxiety, and chronic liver disease), and physiological measures or biomarkers (blood pressure, total cholesterol, high-density lipoprotein and low-density lipoprotein cholesterol, triglycerides, estimated glomerular filtration rate, and hemoglobin A_1c_). Race and ethnicity was included as a potential confounding factor in this study as the prevalence of AMD varies among different racial and ethnic groups.^16^ Comorbid conditions were defined by coded diagnoses recorded in CPRD GOLD and CPRD Aurum. Physiological and biomarker measurements were categorized based on clinically meaningful thresholds (such as high, normal, and low levels; Table 1) according to commonly used clinical guidelines in the UK. The absence of a record of any diagnosis or prescription was taken to indicate the absence of these conditions or medications, respectively. For cohort 1, memantine prescription at baseline was also included as a covariate. For cohorts 2 and 3, the number of preexisting prescription counts of first-line dementia pharmacologic treatments and the preexisting prescription of each first-line dementia pharmacologic treatment were included as covariates.

**Table 1.** Baseline Sociodemographic Characteristics, Behavioral or Lifestyle Risk Factors, Dementia-Related Characteristics, Comorbidities, and Metabolic Characteristics or Biomarkers^a^

Individuals with dementia	Cohort 1		Cohort 2	
	Donepezil (n = 104 237)	Rivastigmine or galantamine (n = 85 791)	Memantine (n = 58 344)	Donepezil, rivastigmine, or galantamine (n = 27 700)
**Sociodemographic characteristics**				
Age, mean (SD), y	80.4 (7.7)	80.5 (7.6)	81.4 (7.8)	80.4 (7.7)
Sex, No. (%)				
Male	38 308 (36.8)	31 952 (37.2)	24 037 (41.2)	11 014 (40.1)
Female	65 929 (63.2)	53 839 (62.9)	34 307 (58.8)	16 627 (59.9)
Race and ethnicity, No. (%)				
Black	2293 (2.2)	1800 (2.1)	1193 (2.0)	592 (2.1)
South Asian	1574 (1.5)	1202 (1.4)	934 (1.6)	401 (1.4)
White	57 711 (55.4)	45 066 (52.5)	34 616 (59.3)	15 778 (57.0)
Mixed race	193 (0.2)	134 (0.2)	101 (0.2)	50 (0.2)
Other^b^	575 (0.6)	433 (0.5)	292 (0.5)	144 (0.5)
Missing	41 891 (40.2)	37 157 (43.4)	21 208 (36.3)	10 737 (38.8)
**Behavioral or lifestyle risk factors**				
BMI, mean (SD)	25.5 (4.6)	25.5 (4.9)	25.6 (4.8)	25.4 (4.6)
Smoking status, No. (%)				
Nonsmoker	40 811 (39.2)	33 823 (39.4)	21 743 (37.3)	11 088 (40.0)
Ex-smokers	44 426 (42.6)	35 607 (41.5)	27 034 (46.3)	12 313 (44.4)
Current smokers	16 743 (16.1)	13 832 (16.1)	8765 (15.0)	3881 (14.0)
Missing	2257 (2.2)	2530 (2.9)	802 (1.4)	419 (1.5)
Drinking status, No. (%)				
Nondrinker	10 974 (10.5)	9578 (11.2)	6357 (10.9)	3198 (11.5)
Ex-drinkers	5503 (5.3)	4396 (5.1)	3890 (6.7)	1771 (6.4)
Current drinkers	80 043 (76.8)	64 491 (75.2)	43 437 (74.4)	20 375 (73.6)
Missing	7717 (7.4)	7327 (8.5)	4660 (8.0)	2358 (8.5)
**Dementia-related characteristics**				
Dementia duration, median (IQR), y	0.3 (0.1-0.7)	1.0 (0.6-1.7)	1.0 (0.3-2.8)	2.0 (0.5-4.4)
Vascular dementia, No. (%)	8342 (8.0)	7985 (9.3)	8051 (13.8)	2699 (9.7)
Nonvascular dementia, No. (%)	61 141 (58.7)	56 212 (65.5)	44 208 (75.8)	23 833 (86.0)
Alzheimer disease, No. (%)	72 667 (69.7)	56 650 (66.0)	38 430 (65.9)	19 937 (72.0)
**Comorbidities, No. (%)**				
Hypertension	56 840 (54.5)	45 663 (53.2)	33 059 (56.7)	14 615 (52.8)
Chronic kidney disease	23 269 (22.3)	18 692 (21.8)	15 290 (26.2)	6509 (23.5)
Ischemic heart disease	18 622 (17.9)	15 676 (18.3)	13 379 (22.9)	5349 (19.3)
Stroke or TIA	12 373 (11.9)	10 750 (12.5)	9154 (15.7)	3782 (13.7)
Myocardial infarction	6234 (6.0)	5396 (6.3)	4988 (8.5)	1884 (6.8)
Heart failure	5633 (5.4)	4793 (5.6)	4790 (8.2)	1643 (5.9)
Atrial fibrillation	10 573 (10.1)	8916 (10.4)	9144 (15.7)	3113 (11.2)
Peripheral vascular disease	2624 (2.5)	2198 (2.6)	1703 (2.9)	673 (2.4)
Aortic atheroma and plaque	3763 (3.6)	3175 (3.7)	2696 (4.6)	1091 (3.9)
Type 1 diabetes	655 (0.6)	500 (0.6)	402 (0.7)	166 (0.6)
Type 2 diabetes	15 850 (15.2)	12 635 (14.7)	10 009 (17.2)	4246 (15.3)
Peripheral neuropathy	8042 (7.7)	6188 (7.2)	4787 (8.2)	2057 (7.4)
Diabetic retinopathy				
No diabetic retinopathy	98 783 (94.8)	81 601 (95.1)	54 737 (93.8)	26 212 (94.6)
Background diabetic retinopathy	3703 (3.6)	2829 (3.3)	2441 (4.2)	1011 (3.7)
STDR	1751 (1.7)	1361 (1.6)	1166 (2.0)	477 (1.7)
Diabetic foot disease				
No diabetic foot	90 863 (87.2)	75 634 (88.2)	49 177 (84.3)	24 058 (86.8)
Diabetic foot	12 250 (11.8)	9231 (10.7)	8485 (14.5)	3337 (12.0)
Diabetic foot ulcer	1124 (1.1)	926 (1.1)	682 (1.2)	306 (1.1)
Osteoporosis	46 964 (45.1)	37 892 (44.2)	27 244 (46.7)	12 482 (45.1)
Osteoarthritis	15 093 (14.5)	12 017 (14.0)	9616 (16.5)	4491 (16.2)
Rheumatoid arthritis	2258 (2.2)	1966 (2.3)	1300 (2.2)	97 (0.3)
Gout	6769 (6.5)	5406 (6.3)	4366 (7.5)	1835 (6.6)
Hypothyroidism	12 277 (11.8)	10 251 (11.9)	7293 (12.5)	3354 (12.1)
Hyperthyroidism	2557 (2.5)	2079 (2.4)	1498 (2.6)	709 (2.6)
Depression	24 945 (23.9)	21 138 (24.6)	15 572 (26.7)	7565 (27.3)
Anxiety	18 780 (18.0)	15 507 (18.1)	12 053 (20.7)	5813 (21.0)
Chronic liver disease	431 (0.4)	363 (0.4)	267 (0.5)	107 (0.4)
**Metabolic characteristics or biomarkers**				
Systolic BP, mm Hg				
<140	63 986 (61.4)	52 655 (61.4)	40 375 (69.2)	19 528 (70.5)
≥140	39 535 (37.9)	32 539 (37.9)	17 710 (30.4)	8023 (29.0)
Missing	716 (0.7)	597 (0.7)	259 (0.4)	149 (0.5)
Diastolic BP, mm Hg				
<90	97 043 (93.1)	79 682 (92.9)	55 077 (94.4)	26 251 (94.8)
≥90	6463 (6.2)	5497 (6.4)	3003 (5.1)	1297 (4.7)
Missing	731 (0.7)	613 (0.7)	264 (0.5)	153 (0.6)
Total cholesterol, mg/dL				
<200.8	52 838 (50.7)	41 769 (48.7)	33 920 (58.1)	15 253 (55.1)
200.8-235.4	20 629 (19.8)	16 590 (19.3)	10 348 (17.7)	5087 (18.4)
≥235.5	16 003 (15.4)	13 433 (15.7)	7310 (12.5)	3729 (13.5)
Missing	14 767 (14.2)	13 999 (16.3)	6766 (11.6)	3632 (13.1)
HDL cholesterol, mg/dL				
<59.1	43 640 (41.9)	33 977 (39.6)	28 369 (48.6)	12 948 (46.7)
≥59.1	40 941 (39.3)	32 852 (38.3)	22 134 (37.9)	10 598 (38.3)
Missing	19 656 (18.9)	18 962 (22.1)	7841 (13.4)	4155 (15.0)
LDL cholesterol, mg/dL				
<100.4	30 224 (29.0)	22 606 (26.3)	21 291 (36.5)	9321 (33.6)
100.4-131.1	17 672 (17.0)	13 996 (16.3)	10 195 (17.5)	4790 (17.3)
131.2-162.1	11 578 (11.1)	9336 (10.9)	6277 (10.8)	3123 (11.3)
162.2-189.1	4795 (4.6)	3865 (4.5)	2354 (4.0)	1183 (4.3)
≥189.2	2321 (2.2)	2025 (2.4)	1159 (2.0)	612 (2.2)
Missing	37 647 (36.1)	33 964 (39.6)	17 068 (29.3)	8672 (31.3)
Triglycerides, mg/dL				
<149.6	60 590 (58.1)	46 773 (54.5)	35 962 (61.6)	16 506 (59.6)
149.6-199.9	11 278 (10.8)	9141 (10.7)	6976 (12.0)	3349 (12.1)
200.0-498.1	7464 (7.2)	5891 (6.9)	4503 (7.7)	2130 (7.7)
≥498.2	145 (0.1)	104 (0.1)	89 (0.2)	42 (0.2)
Missing	24 760 (23.8)	23 881 (27.8)	10 814 (18.5)	5673 (20.5)
eGFR, %				
<30	939 (0.9)	965 (1.1)	497 (0.9)	269 (1.0)
30-59	9402 (9.0)	7001 (8.2)	6597 (11.3)	3087 (11.1)
60-89	16 059 (15.4)	13 328 (15.5)	10 206 (17.5)	5252 (19.0)
≥90	2007 (1.9)	1246 (1.5)	1168 (2.0)	537 (1.9)
Missing	75 830 (72.7)	63 253 (73.7)	39 876 (68.3)	18 556 (67.0)
HbA_1c_, %				
<5.7	10 305 (9.9)	7228 (8.4)	6705 (11.5)	3280 (11.8)
5.7-6.4	11 478 (11.0)	8196 (9.6)	7668 (13.1)	3532 (12.7)
6.5-7.4	4705 (4.5)	3570 (4.2)	2782 (4.8)	1188 (4.3)
≥7.5	3240 (3.1)	2664 (3.1)	2027 (3.5)	864 (3.1)
Missing	74 509 (71.5)	64 134 (74.8)	39 162 (67.1)	18 836 (68.0)

Abbreviations: BMI, body mass index (calculated as weight in kilograms divided by height in meters squared); BP, blood pressure; eGFR, estimated glomerular filtration rate; HbA_1c_, glycated hemoglobin; HDL, high-density lipoprotein; LDL, low-density lipoprotein; STDR, sight-threatening diabetic retinopathy; TIA, transient ischemic attack.

SI conversion factors: To convert total cholesterol, HDL cholesterol, and LDL cholesterol to millimoles per liter, multiply by 0.0259; triglycerides to millimoles per liter, multiply by 0.0113; and HbA_1c_ to proportion of total hemoglobin, multiply by 0.01.

^a^ Data are presented for the first multiple imputed standardized mortality ratio–weighted cohort.

^b^ Other includes any race or ethnicity that did not fall into the categories of Black, South Asian, White, or mixed race (ie, individuals with ≥2 races or ethnicities).

### Weighting

For each of the 3 cohorts, the standardized mortality ratio (SMR) weighting method was applied as the exposure model to investigate the mean treatment effect in the treated population. Multiple imputation by groups (using predictive mean matching) was used for all variables with less than or equal to 20% missing values, with 5 multiply imputed complete datasets being created, under the assumption of missing at random. Missingness in race and ethnicity was treated as a separate category. Initially, logistic regression models were used to obtain PSs, the conditional probability of treatment given all confounders. Standardized mortality ratio weighting involves setting weights to 1 for the exposed patients and PS/(1 − PS) for the unexposed patients. After removing nonoverlapping SMR values, any extreme weight lower than 1% or higher than 99% quantile was trimmed and imputed with either 1% or 99% quantile value, respectively.

### Statistical Analysis

Statistical analysis was carried out between February and November 2023. Baseline characteristics of individuals in the exposed and comparator groups were reported using appropriate descriptive statistics (mean or median for continuous variables with a normal or skewed distribution and proportions for categorical variables). Crude incidence rates per 1000 person-years for the outcome of interest were estimated for the exposed and comparator groups using Poisson regression models.

Competing Cox proportional hazards regression models were used to calculate the crude and adjusted hazard ratios (HRs), together with their corresponding 95% CIs, for development of the outcome in the exposed group compared with the comparator group. Death during the follow-up period was treated as a competing event. In a sensitivity analysis, time-dependent PS matching involved all covariates, followed by competing Cox proportional hazards regression models. For all covariates with missing values, missingness was treated as a separate category in the Cox proportional hazards regression models. The results derived from all 5 multiply imputed, weighted complete datasets were combined using the Rubin rules to produce estimates and 95% CIs that incorporate missing-data uncertainty. Analyses were performed separately for CPRD GOLD and CPRD Aurum and pooled using individual patient data meta-analysis.

All statistical tests were 2-tailed, and *P* < .05 was considered statistically significant. R, version 4.2.2 (R Project for Statistical Computing) was used throughout the data preprocessing and analysis process.

## Results

### Study Population and Baseline Characteristics

There were 132 846 individuals included in cohort 1 (104 237 donepezil users vs 28 609 rivastigmine or galantamine users); the mean (SD) age was 80.4 (7.6) years; 82 132 (61.8%) were female, and 50 714 (38.2%) were male; the mean (SD) BMI was 25.5 (4.6); and 21 078 (15.9%) were current smokers (eTable 4 in Supplement 1). There were 159 419 individuals included in cohort 2 (58 344 memantine users vs 101 075 donepezil, rivastigmine, or galantamine users); the mean (SD) age was 81.2 (7.6) years; 95 237 (59.7%) were female, and 64 182 (40.3%) were male; the mean (SD) BMI was 25.6 (4.7); and 23 956 (15.0%) were current smokers (eTable 5 in Supplement 1). The Figure shows the flowchart for the participant selection procedure for cohorts 1 and 2. In the sensitivity analysis, cohort 3, 92 328 participants were included (58 568 memantine users vs 33 760 rivastigmine or galantamine users); the mean (SD) age was 80.9 (7.7) years; 54 022 (58.5%) were female and 38 306 (41.5%) were male; the mean (SD) BMI was 25.5 (4.7); and 13 767 (14.9%) were current smokers (eFigure 2 in Supplement 1).

**Figure.**
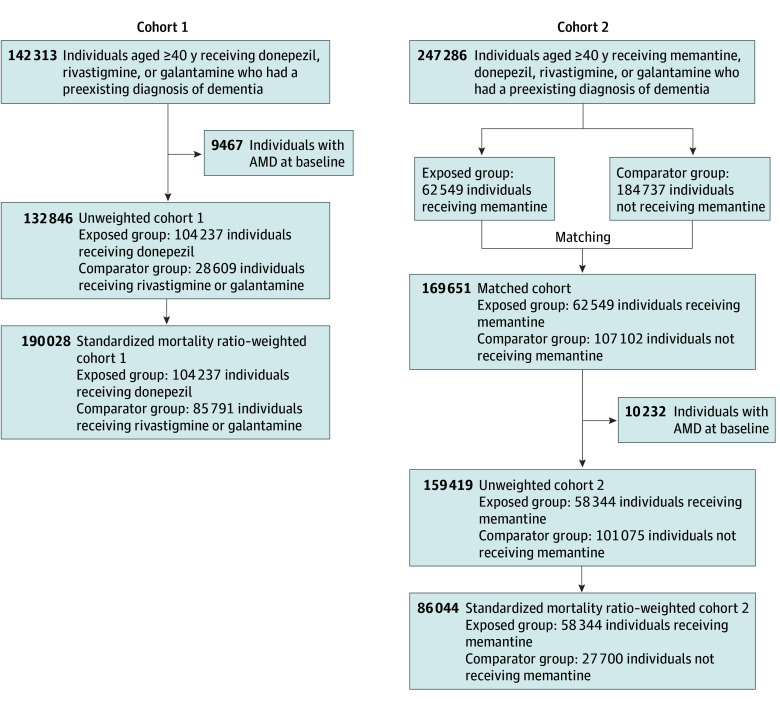
Flowchart for the Participant Selection Procedure for Cohorts 1 and 2 AMD indicates age-related macular degeneration.

In the unweighted cohort 1 (eTable 4 in Supplement 1), there was a lower proportion of men in the exposed group (donepezil) than in the comparator group (rivastigmine or galantamine) (38 308 of 104 237 [36.8%] vs 12 406 of 28 609 [43.4%]); a higher proportion of patients with Alzheimer disease (72 667 of 104 237 [69.7%] vs 14 743 of 28 609 [51.5%]) and a lower proportion of patients with other dementia (61 141 of 104 237 [58.7%] vs 24 124 of 28 609 [84.3%]); a slightly shorter dementia duration (median, 0.3 years [IQR, 0.1-0.7 years] vs 1.4 years [IQR, 0.8-2.1 years]); and a lower prevalence of comorbidities. eTable 5 in Supplement 1 shows the baseline characteristics of cohort 2 before weighting; there was a lower proportion of patients in the exposed group (memantine) than in the comparator group (donepezil, rivastigmine, or galantamine) with vascular dementia (8051 of 58 344 [13.8%] vs 8357 of 101 075 [8.3%]) and other dementia (44 208 of 58 344 [75.8%] vs 72 495 of 101 075 [71.7%]), a lower proportion of patients with Alzheimer disease (38 430 of 58 344 [65.9%] vs 70 089 of 101 075 [69.3%]), and a higher prevalence of comorbidities.

After SMR weighting, the imbalance between exposed and comparator groups was largely mitigated (Table 1). The baseline characteristics for the PS-matched cohorts are presented in eTable 6 in Supplement 1.

### Donepezil and AMD

Over a median follow-up time of 2.2 years (IQR, 1.0-4.0 years), 1167 of 104 237 individuals (1.1%) in the exposed group prescribed donepezil and 205 of 28 609 individuals (0.7%) in the comparator group prescribed rivastigmine or galantamine developed AMD during the study period (cohort 1), corresponding to a crude incidence rate of 3.8 (95% CI, 3.6-4.0) and 3.3 (95% CI, 2.9-3.8) per 1000 person-years, respectively.

After SMR weighting, 1167 of 104 237 individuals (1.1%) in the exposed group and 696 of 85 791 individuals (0.8%) in the comparator group received a diagnosis of AMD, corresponding to crude incidence rates of 3.8 (95% CI, 3.6-4.0) and 3.6 (95% CI, 3.3-4.0) per 1000 person-years, respectively (Table 2). After adjusting for potential confounders, no statistically significant difference in the risk of developing AMD was observed in the donepezil group compared with the rivastigmine or galantamine group among individuals with dementia (adjusted HR, 0.95 [95% CI 0.67-1.35]).

**Table 2.** Risk of Developing Age-Related Macular Degeneration Among Donepezil or Memantine Users and Comparator Drug Users^a^

Characteristic	Cohort 1		Cohort 2		Cohort 3	
	Donepezil (n = 104 237)	Rivastigmine or galantamine (n = 85 791)	Memantine (n = 58 344)	Donepezil, rivastigmine, or galantamine (n = 27 700)	Memantine (n = 58 568)	Rivastigmine or galantamine (n = 6535)
No. of events	1167	696	426	168	428	51
No. of person-years	310 086.1	19 320.5	115 256.7	55 758.0	115 754.5	14 407.0
Incident rate (95% CI)^b^	3.8 (3.6-4.0)	3.6 (3.3-4.0)	3.7 (3.4-4.1)	3.0 (2.5-3.5)	3.7 (3.4-4.1)	2.9 (1.9-4.3)
Model 1, crude HR (95% CI)	1.01 (0.72-1.44)	1 [Reference]	1.20 (0.99-1.44)	1 [Reference]	1.19 (0.87-1.63)	1 [Reference]
Model 2, djusted HR (95% CI)^c^	0.95 (0.67-1.35)	1 [Reference]	1.03 (0.83-1.27)	1 [Reference]	1.24 (0.83-1.86)	1 [Reference]
Model 3, adjusted HR (95% CI)^d^	0.93 (0.66-1.33)	1 [Reference]	1.15 (0.66-1.98)	1 [Reference]	1.10 (0.60-1.90)	1 [Reference]

Abbreviation: HR, hazard ratio.

^a^ Data in rows 2 to 4 are presented for the first multiple imputed standardized mortality ratio–weighted cohort, while data in rows 5 to 7 were combined estimates of 5 imputed datasets.

^b^ Per 1000 person-years.

^c^ Standardized mortality ratio–weighted analyses adjusted for age, sex, body mass index categories, race and ethnicity, index year, data source, smoking status, drinking status, dementia duration, vascular dementia, other dementia, Alzheimer disease, memantine use (cohort 1 only), preceding comparator drug prescription counts (cohort 2 or 3 only), hypertension, chronic kidney disease, ischemic heart disease, stroke or transient ischemic attack, myocardial infarction, heart failure, atrial fibrillation, peripheral vascular disease, aortic atheroma and plaque, type 1 diabetes, type 2 diabetes, peripheral neuropathy, diabetic retinopathy, diabetic foot, osteoporosis, osteoarthritis, rheumatoid arthritis, gout, hypothyroidism, hyperthyroidism, depression, anxiety, chronic liver disease, systolic blood pressure, diastolic blood pressure, total cholesterol, high-density lipoprotein cholesterol, low-density lipoprotein cholesterol, triglycerides, and glycated hemoglobin.

^d^ Propensity score–matched analyses.

### Memantine and AMD

During the study period, over a median follow-up time of 1.5 years (IQR, 0.7-2.8 years), there were 426 AMD events (0.7%) among 58 344 individuals in the exposed group prescribed memantine and 805 AMD events (0.8%) among 101 075 individuals in the comparator group prescribed donepezil, rivastigmine, or galantamine (cohort 2), corresponding to a crude incidence rate of 3.7 (95% CI, 3.4-4.1) and 3.9 (95% CI, 3.6-4.2) per 1000 person-years, respectively.

In the SMR-weighted cohort 2, 426 of 58 344 individuals (0.7%) in the exposed group and 168 of 27 700 individuals (0.6%) in the comparator group received a diagnosis of AMD, which translated to a crude incidence rate of 3.7 (95% CI, 3.4-4.1) and 3.0 (95% CI, 2.5-3.5) per 1000 person-years, respectively (Table 2). In the exposed group compared with the comparator group, the adjusted HR for AMD was 1.03 (95% CI, 0.83-1.27).

### Sensitivity Analyses

The results of the cohort 3 sensitivity analysis, in which individuals prescribed memantine were compared with those prescribed rivastigmine or galantamine only, were broadly similar to the results of cohort 2. After SMR weighting, 428 AMD events (0.7%) among 58 568 individuals in the exposed group (memantine) and 51 events (0.8%) among 6535 individuals in the comparator group (rivastigmine or galantamine) were observed, which translated to an adjusted HR of 1.24 (95% CI, 0.83-1.86).

The results of the PS matching were consistent with the results obtained using SMR weighting in all 3 cohorts (Table 2). The results of all 3 cohorts specifically for CPRD GOLD and CPRD Aurum are presented in eTable 7 in Supplement 1.

## Discussion

In these 3 population-based cohort studies, the association between pharmacologic treatments for dementia and the risk of developing AMD was investigated for the first time, to our knowledge. The crude incidence rate for the development of AMD was higher among individuals prescribed donepezil compared with those prescribed rivastigmine or galantamine and among those prescribed memantine compared with donepezil, rivastigmine, or galantamine or rivastigmine or galantamine. However, after adjustment for potential confounders, the adjusted HRs revealed no statistically significant difference between either donepezil and other first-line dementia drugs, or between memantine (a second-line therapy) and any first-line dementia drugs.

The pathophysiological process leading to AMD is not fully explained, although some mechanisms leading to the dysfunction and degeneration of the retinal pigment epithelium have been proposed. The degeneration of the retinal pigment epithelium observed during aging is a result of accumulative oxidative stress and chronic inflammation.^17,18^ The dysfunction in proteostasis and lipid homeostasis as well as in the level of mitochondria is part of the procedure, whereby the retinal pigment epithelium is unable to regulate all the waste products (proteins and abnormal lipids), which are accumulated and result in the formulation of drusen.^19^ From the level of drusen formulation until the manifestation of AMD and blindness, many other pathophysiological abnormalities may occur.^18^ For example, the increased oxidative stress of the retina during aging is associated with increased levels of reactive oxygen and nitrogen species, both of which activate inflammation and accelerate cellular damage and death in the retina.^20^ Inflammation is also present during the development of AMD at the level of drusen formation, as their products attract inflammatory cells and activate the nucleotide-binding oligomerization domain–like receptor protein-3 inflammasomes and caspase-1, leading to the production of classic inflammatory cytokines and activation of retina cell death.^21,22^ Hence, stopping or decelerating the inflammation process and decreasing oxidative stress may be key to controlling the development and manifestation of AMD.

Taking into account these inflammatory pathways, Bardak et al^23^ investigated the association of memantine with 2-ethylpyridine–induced oxidative stress and mitochondrial dysfunction in human retinal pigment epithelial cells in vitro. The results showed that memantine decreased the activities of caspase-3 and -9, as well as the expression of procaspase and poly(ADP-ribose) polymerase, decreasing further cell apoptosis. Similarly, Neekhra et al^24^ showed that the use of memantine protected human retinal pigment epithelial cells from 7-ketocholesterol–induced apoptosis, by decreasing the activation of caspase-3/7, -8, and -12. These results indicate a possible protective role in the development of AMD. Because other dementia medications, such as donepezil, galantamine, and rivastigmine, also have antioxidant activity, further research is needed to investigate a possible in vitro and in vivo role in AMD.^9,25,26^

However, clinical evidence for the prevention or treatment of AMD using dementia drugs is lacking. To our knowledge, this is the first study examining the association between widely used dementia medications and the development of AMD. Our adjusted results showed no statistically significant association of donepezil and other first-line medications or memantine and other dementia medications with the development of AMD. After a systematic search, we found only 2 other studies examining these drugs and their association with AMD. Wang et al^11^ screened more than 4000 drugs in a nested case-control study to identify if any of these drugs were positively associated with the development or delay of neovascular AMD. All dementia medications were found to have a statistically significant protective association, particularly donepezil, which showed a beneficial association after only a relatively short duration of exposure (<6 months). Similarly, when Alzheimer disease was treated with acetylcholinesterase inhibitors in a large cohort of veterans, there was a small but statistically significant decrease in the long-term risk of AMD.^27^ If the comparator drugs used in our study also have a protective association, that may explain the lack of association observed. However, data from randomized clinical trials revealed no difference in the progression of glaucoma when patients were treated with memantine compared with placebo.^28^ Furthermore, much of the evidence on the neuroprotective associations of dementia medications is focused on Alzheimer disease and may be limited to certain neuronal cell populations and pathways that are not directly or significantly relevant to the biology of the neural retina. It is also possible that the protection may be due to off-target (ie, independent of cholinesterase inhibition) activities of dementia medications, including against amyloid-β toxic effects, glutamate-induced neurotoxic effects, neuroinflammation and tau pathologic characteristics, and vascular pathologic characteristics—which may or may not play a role in the cause of AMD.

### Implications

With regard to implications for clinical practice, our results found no significant difference in the risk of AMD among patients prescribed any of the dementia medications examined, indicating no differences in safety with respect to the development of AMD among patients with dementia. Despite our negative findings, given the potential limitations, further in vitro and in vivo research is recommended to examine any possible pathophysiological protective action of memantine and other dementia medications against the development of AMD, to assess whether this supports a future randomized clinical trial. Given the potential masking of any association by the comparator drugs in this study, if in vitro or in vivo studies were to offer any evidence of a protective association, a randomized clinical trial could then provide a more definitive answer by comparing memantine or other dementia drugs with placebo. In addition, it may be of value to examine the association of these drugs with the progression of AMD, along with the association of combination therapy.

### Strengths and Limitations

This study has some strengths. It is large and well powered, with 3 matched or weighted cohorts, enabling us for the first time to examine the associations between commonly prescribed dementia medications and the development of AMD, compared with other dementia medications. Furthermore, we adjusted our results for a number of potential confounders.

However, there are several limitations to our analysis. Although the study was conducted in a large, racially and ethnically diverse UK-based population, the findings may not be generalizable to other countries with different population characteristics, particularly race and ethnicity, or with different health care systems (eg, those with different diagnostic or treatment pathways for AMD or dementia). Information on the severity of dementia was not available, and therefore it was not possible to directly adjust for this in the analysis. Similarly, information on stage of AMD is poorly recorded in primary health care records, so it was not possible to examine potential differences in the association between dry and wet AMD. The incidence of AMD in our study population was low, which may have prevented the detection of small but clinically meaningful differences in AMD risk between groups. The low incidence may be in part due to the older age of the cohort. Neovascular AMD typically occurs in the mid-to-late 70s, and earlier-stage AMD typically occurs from 50 years of age onward, while the mean age in our cohort was 80 years; there may be an element of survival bias among those included in the cohort, who were required to be free of AMD at the index date. The low AMD incidence may also be in part due to the difficulty in diagnosing AMD among patients with dementia, or these patients not undergoing routine eye examinations, leading to underdiagnosis. It is possible that some participants may have had undiagnosed AMD, either due to early-stage disease or arising from difficulty diagnosing AMD among individuals with dementia; this may have affected the exposed and comparator groups unequally, especially because those prescribed memantine are likely to have more severe or later-stage dementia. This study was conducted in a population with a mean age of 80 years; results may not be generalizable to younger populations. Furthermore, while the CPRD contains data on prescriptions issued, we were not able to ascertain whether the medications were taken by the patients, which may lead to potential misclassification bias. In addition, prescriptions issued in secondary care are not captured, but most prescriptions initiated in secondary care are continued in primary care, and we therefore expect the level of exposure misclassification arising from missed secondary care prescriptions to be low. We controlled for many clinically relevant confounders in both the matching and analysis stages; however, potential unmeasured residual confounding bias and measurement error cannot be completely ruled out. Finally, if all of the dementia medications examined have some protective association for the development of dementia, then any protective association of memantine or donepezil may have been masked by the comparator drugs; however, comparison with a population with untreated dementia would have been subject to confounding arising from significant differences between patients who are treated for dementia or patients who are not treated for dementia.

## Conclusions

In this highly powered cohort study of patients with dementia, we found no statistically significant association between memantine or donepezil and the development of AMD. However, it is important to recognize the limitations of this type of study, such as potential masking of association by the comparator group; therefore, further research is recommended.

## Notes

Supplement 1. **eFigure 1.** Graphic Illustration of the Matching Process in the Prevalent New-User Design **eFigure 2.** Flow Chart of Cohort Three **eTable 1.** Clinical Codes for Dementia **eTable 2.** Clinical Codes for Donepezil, Rivastigmine, Galantamine, and Memantine **eTable 3.** Clinical Codes for Age-Related Macular Degeneration **eTable 4.** Baseline Demographic Characteristics, Behavioural Risk Factors, Dementia Related Characteristics, Comorbidities, and Metabolic Characteristics of Unweighted Cohort One **eTable 5.** Baseline Sociodemographic Characteristics, Behavioural/Lifestyle Risk Factors, Dementia-Related Characteristics, Comorbidities, and Metabolic Characteristics/Biomarkers of Unweighted Cohort Two **eTable 6.** Baseline Sociodemographic Characteristics, Behavioural/Lifestyle Risk Factors, Dementia-Related Characteristics, Comorbidities, and Metabolic Characteristics /Biomarkers of Propensity Score Matched Cohorts (Sensitivity Analyses) **eTable 7.** Risk of Developing Age-Related Macular Degeneration in Donepezil/Memantine Users and Comparator Drug Users in CPRD GOLD and CPRD Aurum

Supplement 2.
Data Sharing Statement
