# Supplementary material for: Pharmacologic Treatments for Dementia and the Risk of Developing Age-Related Macular Degeneration
Source: JAMA Netw Open. 2024 Oct 24;7(10):e2441166. doi: 10.1001/jamanetworkopen.2024.41166 (PMC11581610; doi:10.1001/jamanetworkopen.2024.41166)
Supplement: Supplement 1. — eFigure 1. Graphic Illustration of the Matching Process in the Prevalent New-User Design eFigure 2. Flow Chart of Cohort Three eTable 1. Clinical Codes for Dementia eTable 2. Clinical Codes for Donepezil, Rivastigmine, Galantamine, and Memantine eTable 3. Clinical Codes for Age-Related Macular Degeneration eTable 4. Baseline Demographic Characteristics, Behavioural Risk Factors, Dementia Related Characteristics, Comorbidities, and Metabolic Characteristics of Unweighted Cohort One eTable 5. Baseline Sociodemographic Characteristics, Behavioural/Lifestyle Risk Factors, Dementia-Related Characteristics, Comorbidities, and Metabolic Characteristics/Biomarkers of Unweighted Cohort Two eTable 6. Baseline Sociodemographic Characteristics, Behavioural/Lifestyle Risk Factors, Dementia-Related Characteristics, Comorbidities, and Metabolic Characteristics /Biomarkers of Propensity Score Matched Cohorts (Sensitivity Analyses) eTable 7. Risk of Developing Age-Related Macular Degeneration in Donepezil/Memantine Users and Comparator Drug Users in CPRD GOLD and CPRD Aurum [file jamanetwopen-e2441166-s001.pdf]

Supplementary Online Content

Wang J, Antza C, Lee WH, et al. Pharmacologic treatments for dementia and the risk of developing age-related macular degeneration. *JAMA Netw Open.* 2024;7(10):e2441166. doi:10.1001/jamanetworkopen.2024.41166

eFigure 1. Graphic Illustration of the Matching Process in the Prevalent New-User Design

eFigure 2. Flow Chart of Cohort Three

eTable 1. Clinical Codes for Dementia

eTable 2. Clinical Codes for Donepezil, Rivastigmine, Galantamine, and Memantine

eTable 3. Clinical Codes for Age-Related Macular Degeneration

eTable 4. Baseline Demographic Characteristics, Behavioural Risk Factors, Dementia Related Characteristics, Comorbidities, and Metabolic Characteristics of Unweighted Cohort One

eTable 5. Baseline Sociodemographic Characteristics, Behavioural/Lifestyle Risk Factors, Dementia-Related Characteristics, Comorbidities, and Metabolic Characteristics/Biomarkers of Unweighted Cohort Two

eTable 6. Baseline Sociodemographic Characteristics, Behavioural/Lifestyle Risk Factors, Dementia-Related Characteristics, Comorbidities, and Metabolic Characteristics /Biomarkers of Propensity Score Matched Cohorts (Sensitivity Analyses)

eTable 7. Risk of Developing Age-Related Macular Degeneration in Donepezil/Memantine Users and Comparator Drug Users in CPRD GOLD and CPRD Aurum

This supplementary material has been provided by the authors to give readers additional information about their work.

© 2024 Wang J et al. *JAMA Network Open.*

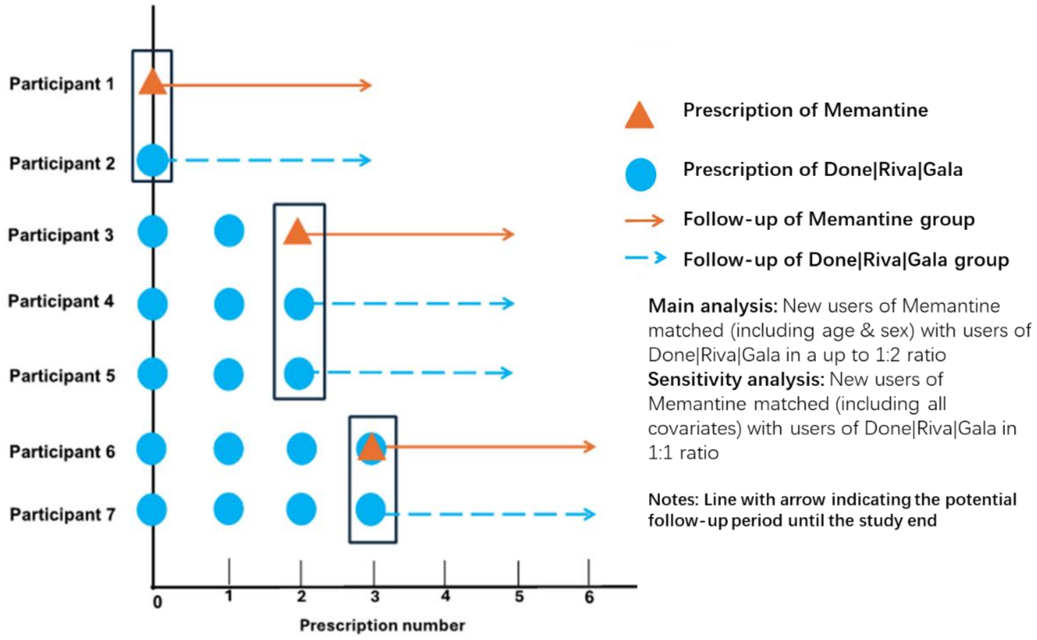

eFigure 1. Graphic Illustration of the Matching Process in the Prevalent New-User Design

For Cohort Two, which uses a prevalent-new user design (1), we have applied time-dependent propensity score matching. We firstly defined exposure sets based on the number of comparator drug prescriptions. Time-conditional propensity scores were constructed using conditional logistic regression with a caliper width of 0.2, stratified by exposure set (Main analysis: age and sex were included; sensitivity analysis: all relevant covariates were included), to predict the probability of receiving Memantine compared to comparator drugs. Within each exposure set, individuals using comparator drugs (unexposed group) were matched without replacement to individuals using Memantine (exposed group), based on the nearest time-conditional propensity score and chronological order. This matching process ensured that people in both exposed group and comparator group had the similar possibility of having the prescription of Memantine in the same period.

eReference.

1. Suissa S, Moodie EE, Dell'Aniello S. Prevalent new-user cohort designs for comparative drug effect studies by time-conditional propensity scores. Pharmacoepidemiology and drug safety. 2017;26(4):459-468.

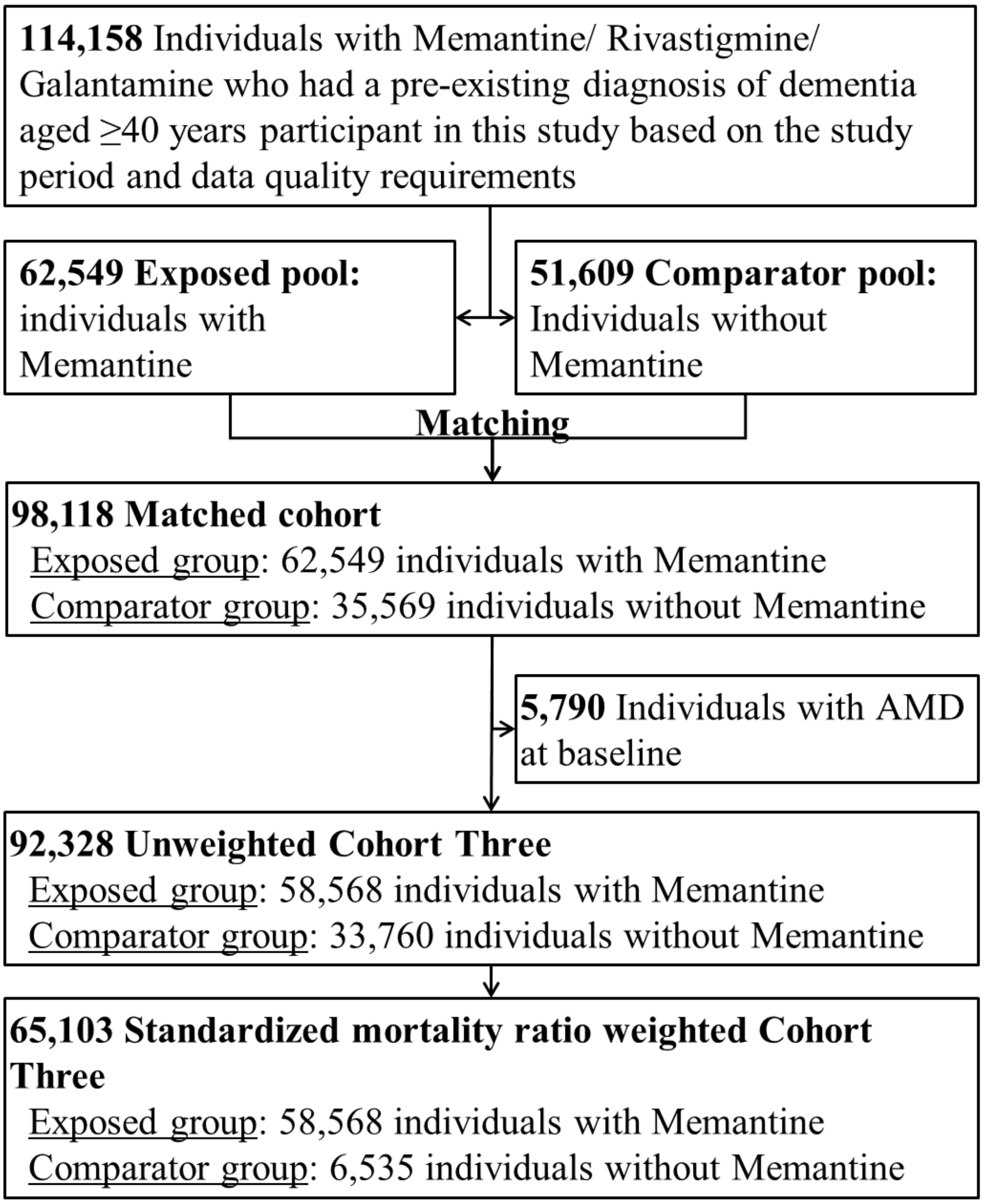

eFigure 2. Flow Chart of Cohort Three

© 2024 Wang J et al. *JAMA Network Open.*

eTable 1. Clinical Codes for Dementia

DESCRIPTION	READ CODE	SNOMED-CT CODE	DATABASE
Senile/presenile dementia	E00..12		GOLD
Senile dementia	E00..11		GOLD
Alzheimer's disease	F110.00		GOLD
[X] Senile dementia NOS	Eu02z14		GOLD
[X] Unspecified dementia	Eu02z00		GOLD
Binswanger's disease	F21y200		GOLD
H/O: dementia	1461		GOLD
[X]Vascular dementia	Eu01.00		GOLD
Uncomplicated senile dementia	E000.00		GOLD
Lewy body disease	F116.00		GOLD
[X]Dementia in Alzheimer's disease	Eu00.00		GOLD
[X]Alzheimer's dementia unspec	Eu00z11		GOLD
Multi infarct dementia	E004.11		GOLD
[X]Subcortical vascular dementia	Eu01200		GOLD
[X]Dementia in Parkinson's disease	Eu02300		GOLD
[X]Arteriosclerotic dementia	Eu01.11		GOLD
Normal pressure hydrocephalus	F113000		GOLD
Pick's disease	F111.00		GOLD
[X]Multi-infarct dementia	Eu01100		GOLD
[X]Senile dementia,Alzheimer's type	Eu00112		GOLD
[X]Dementia in other diseases classified elsewhere	Eu02.00		GOLD
Dementia annual review	6AB..00		GOLD
Presenile dementia	E001.00		GOLD
Alzheimer's disease with early onset	F110000		GOLD
Senile dementia with paranoia	E002000		GOLD
[X]Vascular dementia, unspecified	Eu01z00		GOLD
Arteriosclerotic dementia	E004.00		GOLD
Senile dementia with depression	E002100		GOLD
Korsakoff's non-alcoholic psychosis	E040.11		GOLD
Dementia in conditions EC	E041.00		GOLD
[X]Presenile dementia,Alzheimer's type	Eu00011		GOLD
[X]Lewy body dementia	Eu02500		GOLD
[X]Alcoholic dementia NOS	Eu10711		GOLD
Alcoholic dementia NOS	E012.11		GOLD
Presenile dementia with depression	E001300		GOLD
[X] Senile dementia, depressed or paranoid type	Eu02z16		GOLD
[X]Dementia in Pick's disease	Eu02000		GOLD
[X]Dementia in Alzheimer's disease, unspecified	Eu00z00		GOLD
Presenile dementia with paranoia	E001200		GOLD
Excepted from dementia quality indicators: Patient unsuitable	9hD0.00		GOLD
[X]Dementia in Alzheimer's dis, atypical or mixed type	Eu00200		GOLD
[X]Mixed cortical and subcortical vascular dementia	Eu01300		GOLD
Alzheimer's disease with late onset	F110100		GOLD
[X] Primary degenerative dementia NOS	Eu02z13		GOLD
[X]Dementia in Huntington's disease	Eu02200		GOLD

© 2024 Wang J et al. *JAMA Network Open.*

Senile dementia with delirium	E003.00	GOLD
Jakob-Creutzfeldt disease	A411.00	GOLD
Presenile dementia NOS	E001z00	GOLD
[X]Dementia in Alzheimer's disease with late onset	Eu00100	GOLD
Excepted from dementia quality indicators: Informed dissent	9hD1.00	GOLD
Senile dementia with depressive or paranoid features NOS	E002z00	GOLD
[X]Dementia in human immunodef virus [HIV] disease	Eu02400	GOLD
Arteriosclerotic dementia NOS	E004z00	GOLD
Uncomplicated presenile dementia	E001000	GOLD
Uncomplicated arteriosclerotic dementia	E004000	GOLD
Arteriosclerotic dementia with depression	E004300	GOLD
[X]Primary degen dementia of Alzheimer's type, senile onset	Eu00113	GOLD
Exception reporting: dementia quality indicators	9hD..00	GOLD
Senile dementia with depressive or paranoid features	E002.00	GOLD
[X]Vascular dementia of acute onset	Eu01000	GOLD
[X]Alzheimer's disease type 1	Eu00111	GOLD
[X] Presenile dementia NOS	Eu02z11	GOLD
Cerebral degeneration due to Jakob - Creutzfeldt disease	F11x700	GOLD
[X]Dementia in Alzheimer's disease with early onset	Eu00000	GOLD
Presenile dementia with delirium	E001100	GOLD
Dementia monitoring first letter	9Ou1.00	GOLD
[X]Delirium superimposed on dementia	Eu04100	GOLD
[X]Dementia in Creutzfeldt-Jakob disease	Eu02100	GOLD
Other alcoholic dementia	E012.00	GOLD
Dementia monitoring	66h..00	GOLD
Language disorder of dementia	ZS7C500	GOLD
[X]Other vascular dementia	Eu01y00	GOLD
Arteriosclerotic dementia with paranoia	E004200	GOLD
[X]Predominantly cortical dementia	Eu01111	GOLD
Arteriosclerotic dementia with delirium	E004100	GOLD
[X]Other Alzheimer's disease	Fyu3000	GOLD
[X]Primary degen dementia, Alzheimer's type, presenile onset	Eu00012	GOLD
[X]Alzheimer's disease type 2	Eu00013	GOLD
Drug-induced dementia	E02y100	GOLD
[X]Dementia in other specified diseases classif elsewhere	Eu02y00	GOLD
Dementia monitoring telephone invite	9Ou5.00	GOLD
Binswanger's encephalopathy	F21y211	GOLD
Dementia monitoring second letter	9Ou2.00	GOLD
Dementia monitoring administration	9Ou..00	GOLD
Dementia monitoring third letter	9Ou3.00	GOLD
Dementia monitoring verbal invite	9Ou4.00	GOLD
Frontotemporal degeneration	F118.00	GOLD
Dementia care plan	8CMZ.00	GOLD
Dementia advance care plan agreed	8CSA.00	GOLD
Review of dementia advance care plan	8CMG200	GOLD
Dementia advance care plan declined	8IAe000	GOLD
Antipsychotic drug therapy for dementia	8BPa.00	GOLD

© 2024 Wang J et al. *JAMA Network Open.*

Sporadic Creutzfeldt-Jakob disease	A411000		GOLD
Dementia care plan reviewed	8CMZ100		GOLD
Dementia care plan agreed	8CMZ000		GOLD
Dementia medication review	8BM0200		GOLD
Dementia care plan declined	8CMZ200		GOLD
Dementia advance care plan	8CMe000		GOLD
Dementia advance care plan review declined	8IAe200		GOLD
Dementia care plan review declined	8CMZ300		GOLD
Jakob-Creutzfeldt disease	A411.00	792004	AURUM
Pick's disease	F111.00	13092008	AURUM
Senile dementia	E00..11	15662003	AURUM
Binswanger's disease	F21y200	90099008	AURUM
Uncomplicated presenile dementia	E001000	191451009	AURUM
Senile dementia with depression	E002100	191459006	AURUM
Senile dementia with delirium	E003.00	191461002	AURUM
Uncomplicated arteriosclerotic dementia	E004000	191463004	AURUM
Arteriosclerotic dementia with delirium	E004100	191464005	AURUM
Arteriosclerotic dementia with paranoia	E004200	191465006	AURUM
Arteriosclerotic dementia with depression	E004300	191466007	AURUM
Drug-induced dementia	E02y100	191493005	AURUM
Binswanger's encephalopathy	F21y211	90099008	AURUM
Dementia monitoring administration	9Ou..00	713821000000106	AURUM
Dementia monitoring	66h..00	248711000000102	AURUM
Dementia annual review	6AB..00	249181000000100	AURUM
Other senile/presenile dement.	E00..98	268612007	AURUM
Senile and presenile dementias	E00..99	268612007	AURUM
Senile dementia-acute confused	E003.99	191461002	AURUM
[RFC] Alzheimer's disease		905791000006104	AURUM
[D] Vascular dementia		914921000006101	AURUM
[D] Dementia		914941000006108	AURUM
[D] Dementia in Alzheimer's disease		914951000006105	AURUM
Dementia review		915111000006108	AURUM
Dementia review with third party		915121000006100	AURUM
[RFC] Dementia		939491000006102	AURUM
Variant Creutzfeldt-Jakob disease		304603007	AURUM
History of Creutzfeldt-Jakob disease		1820331000006100	AURUM
Dementia monitoring in secondary care		1856611000006100	AURUM
Dementia medication review		1916981000006100	AURUM
Dementia stage at diagnosis - mid (moderate)		1949641000006100	AURUM
Dementia stage at diagnosis - late (severe)		1949651000006100	AURUM
Person centred dementia support plan (Scotland)		1949681000006100	AURUM
Dementia support plan (Scot) in place at 12 months		1949691000006100	AURUM
Dementia support plan (Scot) partly in place at 12 months		1949701000006100	AURUM
Dementia support plan (Scot) absent at 12 months		1949711000006100	AURUM
Dementia post diagnostic support (PDS) - transition arrangements		1949721000006100	AURUM
Dementia PDS transition - progress to supported self management		1949731000006100	AURUM
Dementia PDS transition - further PDS required		1949751000006100	AURUM

© 2024 Wang J et al. *JAMA Network Open.*

Dementia PDS transition - referred for community care support	1949761000006100	AURUM
Dementia post diagnostic support (PDS)(Scot)- 5 pillar model	1949771000006100	AURUM
Dementia PDS(Scot)-understanding illness/managing symptms status	1950411000006100	AURUM
Dementia PDS understanding illness - pillar not met (pt choice)	1950461000006100	AURUM
Dementia PDS understanding illness - other pillar status	1950481000006100	AURUM
Dementia PDS understanding illness - pillar status not known	1950491000006100	AURUM
Dementia PDS (Scot) planning future care status - pillar met	1950511000006100	AURUM
Dementia PDS planning future care-pillar not met(service reason)	1950531000006100	AURUM
Dementia PDS planning future care - other pillar status	1950551000006100	AURUM
Dementia PDS (Scot) peer support status - pillar met	1950581000006100	AURUM
Dementia PDS (Scot) peer support-pillar not met (service reason)	1950601000006100	AURUM
Dementia PDS (Scot) peer support - pillar not met (pt choice)	1950611000006100	AURUM
Dementia PDS (Scot) peer support - other pillar status	1950621000006100	AURUM
Dementia PDS (Scot) peer support - pillar status not known	1950631000006100	AURUM
Dementia PDS (Scot) - supporting community connections status	1950641000006100	AURUM
Dementia PDS supprt comm. connectns-pillar not met(servce reasn)	1950671000006100	AURUM
Dementia PDS support comm. connectns-pillar not met (pt choice)	1950681000006100	AURUM
Dementia PDS support comm. connectns - other pillar status	1950691000006100	AURUM
Dementia PDS support comm. connectns - pillar status not known	1950701000006100	AURUM
Dementia PDS (Scot) - planning future decision-making status	1950711000006100	AURUM
Dementia PDS planning future decisn-making -pillar partially met	1950731000006100	AURUM
Dementia PDS plan.futur decisn-mkng-pillar not met(servce reasn)	1950741000006100	AURUM
Dementia PDS plan.future decisn-making-pillar not met(pt choice)	1950751000006100	AURUM
Dementia link worker details	1950781000006100	AURUM
Dementia link worker - mental health CPN	1950791000006100	AURUM
No longer has a dementia link worker	1950801000006100	AURUM
Dementia link worker - third sector (Alzheimer Scotland)	1950841000006100	AURUM
Dementia link worker - other third sector worker	1950851000006100	AURUM
Dementia link worker - social worker	1950861000006100	AURUM
Dementia link worker - other	1950871000006100	AURUM
Dementia in Alzheimer's disease with early onset, without additional symptoms	1971401000006100	AURUM
Dementia in Alzheimer's disease with early onset, other symptoms, predominantly hallucinatory	1971771000006100	AURUM
Unspecified dementia, other symptoms, predominantly delusional	1972041000006100	AURUM

© 2024 Wang J et al. *JAMA Network Open.*

Unspecified dementia, other symptoms, predominantly hallucinatory		1972061000006100	AURUM
Unspecified dementia, other symptoms, predominantly depressive		1972071000006100	AURUM
Dementia in Alzheimer's disease with late onset, without additional symptoms		1972171000006100	AURUM
Dementia in Alzheimer's disease with late onset, other symptoms, predominantly delusional		1972181000006100	AURUM
Dementia in Alzheimer's disease with late onset, other symptoms, predominantly hallucinatory		1972191000006100	AURUM
Dementia in Alzheimer's dis, atypical or mixed type, other symptoms, predominantly depressive		1972311000006100	AURUM
Dementia in Alzheimer's dis, atypical or mixed type, other mixed symptoms		1972341000006100	AURUM
Dementia in Alzheimer's disease, unspecified, without additional symptoms		1972371000006100	AURUM
Dementia in Alzheimer's disease, unspecified, other symptoms, predominantly depressive		1972451000006100	AURUM
Vascular dementia of acute onset, other symptoms, predominantly delusional		1972501000006100	AURUM
Vascular dementia of acute onset, other symptoms, predominantly hallucinatory		1972521000006100	AURUM
Vascular dementia of acute onset, other symptoms, predominantly depressive		1972541000006100	AURUM
Vascular dementia of acute onset, other mixed symptoms		1972571000006100	AURUM
Multi-infarct dementia, without additional symptoms		1972601000006100	AURUM
Subcortical vascular dementia, other symptoms, predominantly hallucinatory		1972751000006100	AURUM
Subcortical vascular dementia, other mixed symptoms		1972791000006100	AURUM
Mixed cortical and subcortical vascular dementia, without additional symptoms		1972821000006100	AURUM
Mixed cortical and subcortical vascular dementia, other symptoms, predominantly delusional		1972831000006100	AURUM
Mixed cortical and subcortical vascular dementia, other symptoms, predominantly hallucinatory		1972871000006100	AURUM
Mixed cortical and subcortical vascular dementia, other symptoms, predominantly depressive		1972911000006100	AURUM
Mixed cortical and subcortical vascular dementia, other mixed symptoms		1972931000006100	AURUM
Other vascular dementia, other symptoms, predominantly hallucinatory		1973341000006100	AURUM
Other vascular dementia, other symptoms, predominantly depressive		1973381000006100	AURUM
Vascular dementia, unspecified, without additional symptoms		1973461000006100	AURUM
Vascular dementia, unspecified, other symptoms, predominantly delusional		1973501000006100	AURUM
Vascular dementia, unspecified, other symptoms, predominantly hallucinatory		1973531000006100	AURUM
Antipsychotic drug therapy for dementia	8BPa.00	700214004	AURUM
Sporadic Creutzfeldt-Jakob disease	A411000	713060000	AURUM
Dementia care plan agreed	8CMZ000	956841000000106	AURUM
Dementia care plan reviewed	8CMZ100	956861000000107	AURUM
Dementia care plan declined	8CMZ200	956881000000103	AURUM
Dementia advance care plan	8CMe000	959361000000105	AURUM

© 2024 Wang J et al. *JAMA Network Open.*

Dementia advance care plan review declined	8IAe200	959461000000102	AURUM
Arteriosclerotic dementia	E004.00	56267009	AURUM
Senile dementia with depressive or paranoid features NOS	E002z00	191457008	AURUM
Senile or presenile psychoses NOS	E00z.00	268612007	AURUM
[X]Other vascular dementia	Eu01y00	429998004	AURUM
[X]Dementia in other diseases classified elsewhere	Eu02.00	191519005	AURUM
[X]Dementia in Pick's disease	Eu02000	21921000119103	AURUM
[X]Dementia in Parkinson's disease	Eu02300	425390006	AURUM
[X]Dementia in other specified diseases classif elsewhere	Eu02y00	191519005	AURUM
[X]Other Alzheimer's disease	Fyu3000	26929004	AURUM
Other alcoholic dementia	E012.00	281004	AURUM
Exception reporting: dementia quality indicators	9hD..00	715881000000108	AURUM
Excepted from dementia quality indicators: Informed dissent	9hD1.00	716131000000105	AURUM
[X] Senile dementia NOS	Eu02z14	15662003	AURUM
[X] Unspecified dementia	Eu02z00	52448006	AURUM
[X]Alcoholic dementia NOS	Eu10711	281004	AURUM
[X]Dementia in Alzheimer's disease with early onset	Eu00000	416780008	AURUM
[X]Mixed cortical and subcortical vascular dementia	Eu01300	230287006	AURUM
[X]Multi-infarct dementia	Eu01100	56267009	AURUM
Dementia monitoring verbal invite	9Ou4.00	716221000000104	AURUM
Dementia monitoring telephone invite	9Ou5.00	716991000000108	AURUM
[X]Predominantly cortical dementia	Eu01111	56267009	AURUM
[X]Presenile dementia,Alzheimer's type	Eu00011	416780008	AURUM
[X]Primary degen dementia of Alzheimer's type, senile onset	Eu00113	416975007	AURUM
[X]Primary degen dementia, Alzheimer's type, presenile onset	Eu00012	416780008	AURUM
[X]Subcortical vascular dementia	Eu01200	230286002	AURUM
[X]Vascular dementia	Eu01.00	429998004	AURUM
[X]Vascular dementia of acute onset	Eu01000	230285003	AURUM
Cerebral degeneration due to Jakob - Creutzfeldt disease	F11x700	192818008	AURUM
Lewy body disease	F116.00	80098002	AURUM
Korsakoff's non-alcoholic psychosis	E040.11	17262008	AURUM
Excepted from dementia quality indicators: Patient unsuitabl	9hD0.00	716341000000104	AURUM
Sporadic CJD (Creutzfeldt-Jakob disease)	A411011	713060000	AURUM
Shared care prescribing of drugs for dementia		719787003	AURUM
Arteriosclerotic dementia NOS	E004z00	56267009	AURUM
[X]Dementia in Alzheimer's disease	Eu00.00	26929004	AURUM
[X]Dementia in Alzheimer's disease, unspecified	Eu00z00	26929004	AURUM
[X] Presenile dementia NOS	Eu02z11	12348006	AURUM
[X] Senile dementia, depressed or paranoid type	Eu02z16	191457008	AURUM
[X]Arteriosclerotic dementia	Eu01.11	56267009	AURUM
[X]Dementia in Alzheimer's dis, atypical or mixed type	Eu00200	26929004	AURUM
Dementia care plan	8CMZ.00	736371006	AURUM
Dementia advance care plan agreed	8CSA.00	1095121000000100	AURUM
[X]Senile dementia,Alzheimer's type	Eu00112	416975007	AURUM
Transmissible virus dementia		792004	AURUM
Dementia associated with AIDS		421529006	AURUM

© 2024 Wang J et al. *JAMA Network Open.*

Creutzfeldt-Jakob disease	792004	AURUM
Non-alcoholic Korsakoff's psychosis	17262008	AURUM
Presenile dementia, Alzheimer's type	416780008	AURUM
Primary degenerative dementia of the Alzheimer type, senile onset	416975007	AURUM
Subcortical leukoencephalopathy	90099008	AURUM
Dementia advance care plan agreed	1095121000000100	AURUM
Cerebral degeneration due to Creutzfeldt-Jakob disease	192818008	AURUM
Dementia due to Huntingtons disease	442344002	AURUM
Dementia due to Picks disease	21921000119103	AURUM
ADC - Acquired immune deficiency syndrome dementia complex	421529006	AURUM
Primary degenerative dementia of the Alzheimer type, late onset	416975007	AURUM
Dementia associated with Parkinson Disease	425390006	AURUM
Korsakoff psychosis	69482004	AURUM
Binswanger's dementia	90099008	AURUM
Pick disease	13092008	AURUM
Subcortical arteriosclerotic encephalopathy	90099008	AURUM
Sporadic Jakob-Creutzfeldt disease	713060000	AURUM
Dementia paralytica	51928006	AURUM
SDAT - Senile dementia, Alzheimer's type	416975007	AURUM
NPH - Normal pressure hydrocephalus	30753002	AURUM
Dementia of the Lewy body type	80098002	AURUM
AD - Alzheimer's disease	26929004	AURUM
Alcohol-induced persisting dementia	281004	AURUM
CJD - Creutzfeldt-Jakob disease	792004	AURUM
Nuchal dystonia-dementia syndrome	28978003	AURUM
Creutzfeldt Jakob disease	792004	AURUM
Dementia due to Huntington disease	442344002	AURUM
Dementia of the Alzheimers type with early onset	416780008	AURUM
Dementia co-occurrent and due to Pick's disease	21921000119103	AURUM
Picks disease	13092008	AURUM
Dementia in Alzheimer's disease - type 2	416780008	AURUM
Acquired immune deficiency syndrome dementia complex	421529006	AURUM
LBD - Lewy body disease	80098002	AURUM
Dementia due to Pick disease	21921000119103	AURUM
DLBD - Diffuse Lewy body disease	80098002	AURUM
Primary degenerative dementia of the Alzheimer type, presenile onset	416780008	AURUM
Primary degenerative dementia of the Alzheimer type, early onset	416780008	AURUM
Organic dementia	52448006	AURUM
Lewy body variant of Alzheimer's disease	80098002	AURUM
VAD - Vascular dementia	56267009	AURUM
Wernicke-Korsakoff syndrome	69482004	AURUM
Dementia due to Creutzfeldt-Jakob disease	429458009	AURUM
QOF (Quality and Outcomes Framework) dementia quality indicator-related care invitation	1110901000000100	AURUM
Dementia post diagnostic support (Scotland)	1949611000006100	AURUM
Dementia stage at diagnosis	1949621000006100	AURUM

© 2024 Wang J et al. *JAMA Network Open.*

Dementia stage at diagnosis - not known		1949671000006100	AURUM
Dementia PDS (Scot) understanding illness - pillar partially met		1950431000006100	AURUM
Dementia PDS (Scot) - planning for future care status		1950501000006100	AURUM
Dementia PDS (Scot) planning future care - pillar partially met		1950521000006100	AURUM
Dementia PDS (Scot) peer support status - pillar partially met		1950591000006100	AURUM
Dementia PDS planning future decision-making - pillar met		1950721000006100	AURUM
Dementia link worker - other mental health worker		1950821000006100	AURUM
Unspecified dementia, without additional symptoms		1972021000006100	AURUM
Unspecified dementia, other mixed symptoms		1972081000006100	AURUM
Dementia in Alzheimer's disease with early onset, other symptoms, predominantly depressive		1972131000006100	AURUM
Dementia in Alzheimer's disease with early onset, other mixed symptoms		1972141000006100	AURUM
Dementia in Alzheimer's dis, atypical or mixed type, without additional symptoms		1972231000006100	AURUM
Dementia in Alzheimer's dis, atypical or mixed type, other symptoms, predominantly delusional		1972251000006100	AURUM
Dementia in Alzheimer's disease, unspecified, other mixed symptoms		1972471000006100	AURUM
Vascular dementia of acute onset, without additional symptoms		1972481000006100	AURUM
Multi-infarct dementia, other symptoms, predominantly delusional		1972621000006100	AURUM
Multi-infarct dementia, other symptoms, predominantly depressive		1972661000006100	AURUM
Subcortical vascular dementia, without additional symptoms		1972711000006100	AURUM
Subcortical vascular dementia, other symptoms, predominantly delusional		1972731000006100	AURUM
Other vascular dementia, without additional symptoms		1973221000006100	AURUM
Other vascular dementia, other symptoms, predominantly delusional		1973271000006100	AURUM
Other vascular dementia, other mixed symptoms		1973401000006100	AURUM
Senile dementia - simple type	E000.99	191449005	AURUM
Dementia monitoring first letter	9Ou1.00	715821000000107	AURUM
Dementia monitoring second letter	9Ou2.00	717471000000101	AURUM
Presenile dementia with paranoia	E001200	191454001	AURUM
Dementia in conditions EC	E041.00	191519005	AURUM
[X]Alzheimer's disease type 1	Eu00111	416975007	AURUM
Dementia in Alzheimer's disease - type 1		416975007	AURUM
Cortical Lewy body disease		80098002	AURUM
Subcortical atherosclerotic dementia		90099008	AURUM
Dementia associated with acquired immunodeficiency syndrome		421529006	AURUM
CLBD - Cortical Lewy body disease		80098002	AURUM
AIDS - Acquired immune deficiency syndrome dementia complex		421529006	AURUM
Creutzfeldt-Jakob variant disease		304603007	AURUM
Paralytic dementia		51928006	AURUM
SD - Senile dementia		15662003	AURUM

Non-alcoholic Korsakoff psychosis		17262008	AURUM
Alzheimer dementia		26929004	AURUM
Dementia associated with Parkinson's Disease		425390006	AURUM
Multi infarct dementia		56267009	AURUM
Alzheimer disease		26929004	AURUM
Acquired immune deficiency syndrome-related dementia		421529006	AURUM
[X]Vascular dementia, unspecified	Eu01z00	429998004	AURUM
[X]Dementia in Creutzfeldt-Jakob disease	Eu02100	429458009	AURUM
Signposting to dementia support service		1083391000000100	AURUM
Dementia in Alzheimer's disease with early onset, other symptoms, predominantly delusional		1971541000006100	AURUM
Dementia in Alzheimer's disease with late onset, other symptoms, predominantly depressive		1972201000006100	AURUM
Dementia in Alzheimer's disease with late onset, other mixed symptoms		1972211000006100	AURUM
Dementia in Alzheimer's dis, atypical or mixed type, other symptoms, predominantly hallucinatory		1972291000006100	AURUM
Subcortical vascular dementia, other symptoms, predominantly depressive		1972771000006100	AURUM
Dementia stage at diagnosis - undetermined		1949661000006100	AURUM
Dementia PDS (Scot) understanding illness status - pillar met		1950421000006100	AURUM
Dementia PDS understanding illness-pillar not met(service reasn)		1950451000006100	AURUM
Dementia PDS planning future care - pillar status not known		1950561000006100	AURUM
Dementia PDS (Scot) support community connections - pillar met		1950651000006100	AURUM
Dementia PDS plan future decision-making - other pillar status		1950761000006100	AURUM
Dementia PDS plan future decision-making-pillar status not known		1950771000006100	AURUM
Dementia care plan		1834091000006100	AURUM
Dementia monitoring in primary care		1856601000006100	AURUM
[D] Dementia with Lewy bodies		914931000006103	AURUM
Dementia monitoring third letter	9Ou3.00	716671000000102	AURUM
Alzheimer's disease with early onset	F110000	416780008	AURUM
Alzheimer's disease with late onset	F110100	416975007	AURUM
Uncomplicated senile dementia	E000.00	191449005	AURUM
Presenile dementia with delirium	E001100	191452002	AURUM
Presenile dementia	E001.00	12348006	AURUM
Alzheimer's disease	F110.00	26929004	AURUM
H/O: dementia	1461	161465002	AURUM
[X]Alzheimer's disease type 2	Eu00013	416780008	AURUM
[X]Dementia in Alzheimer's disease with late onset	Eu00100	416975007	AURUM
[X]Dementia in human immunodef virus [HIV] disease	Eu02400	421529006	AURUM
[X]Alzheimer's dementia unspec	Eu00z11	26929004	AURUM
Dementia advance care plan declined	8IAe000	956881000000103	AURUM
Review of dementia advance care plan	8CMG200	956861000000107	AURUM
History of dementia		161465002	AURUM
MID - Multi-infarct dementia		56267009	AURUM
Subcortical leucoencephalopathy		90099008	AURUM

© 2024 Wang J et al. *JAMA Network Open.*

SDLT - Senile dementia of the Lewy body type		80098002	AURUM
Dementia associated with alcoholism		281004	AURUM
[X]Dementia in Huntington's disease	Eu02200	442344002	AURUM
[X]Delirium superimposed on dementia	Eu04100	2776000	AURUM
Dementia medication review	8BM0200	938551000000108	AURUM
Dementia care plan review declined	8CMZ300	956901000000100	AURUM
Shared care prescribing of drugs for dementia declined		720022007	AURUM
Dementia stage at diagnosis - early (mild)		1949631000006100	AURUM
Dementia PDS planning future care - pillar not met (pt choice)		1950541000006100	AURUM
Dementia PDS (Scot) - peer support status		1950571000006100	AURUM
Dementia PDS support community connections -pillar partially met		1950661000006100	AURUM
Dementia link worker - mental health OT		1950811000006100	AURUM
Dementia in Alzheimer's disease, unspecified, other symptoms, predominantly delusional		1972401000006100	AURUM
Dementia in Alzheimer's disease, unspecified, other symptoms, predominantly hallucinatory		1972421000006100	AURUM
Multi-infarct dementia, other symptoms, predominantly hallucinatory		1972641000006100	AURUM
Multi-infarct dementia, other mixed symptoms		1972681000006100	AURUM
Vascular dementia, unspecified, other symptoms, predominantly depressive		1973551000006100	AURUM
Vascular dementia, unspecified, other mixed symptoms		1976831000006100	AURUM
Sporadic Creutzfeldt-Jakob disease		1897891000006100	AURUM
Dementia confirmed		1823871000006100	AURUM
Did not attend dementia monitoring		1856631000006100	AURUM
Refer to dementia care advisor		1774581000006100	AURUM
nvCJD - New variant of Creutzfeldt-Jakob disease		304603007	AURUM
vCJD - variant Creutzfeldt-Jakob disease		304603007	AURUM
Dementia	E00..97	268612007	AURUM
Dementia of the Alzheimers type, late onset		416975007	AURUM
Frontotemporal degeneration	F118.00	230273006	AURUM
Presenile dementia with depression	E001300	191455000	AURUM
Senile dementia with depressive or paranoid features	E002.00	191457008	AURUM
Senile dementia with paranoia	E002000	191458003	AURUM
Normal pressure hydrocephalus	F113000	30753002	AURUM
Alcoholic dementia NOS	E012.11	281004	AURUM
Senile/presenile dementia	E00..12	52448006	AURUM
Multi infarct dementia	E004.11	56267009	AURUM
[X] Primary degenerative dementia NOS	Eu02z13	279982005	AURUM
[X]Lewy body dementia	Eu02500	80098002	AURUM
Presenile dementia NOS	E001z00	12348006	AURUM
JCD - Jakob-Creutzfeldt disease		792004	AURUM

eTable 2. Clinical Codes for Donepezil, Rivastigmine, Galantamine, and Memantine

DONEPEZIL	BNF	DATABASE
Donepezil 5mg tablets (Alliance Healthcare (Distribution) Ltd) Donepezil hydrochloride 5mg Tablet Oral	4110000	GOLD
Donepezil 5mg tablets (Macleods Pharma UK Ltd) Donepezil hydrochloride 5mg Tablet Oral	4110000	GOLD
Donepezil 10mg orodispersible tablets (Consilient Health Ltd) Donepezil hydrochloride 10mg Orodispersible tablet Oral	4110000	GOLD
Donepezil 5mg tablets (Actavis UK Ltd) Donepezil hydrochloride 5mg Tablet Oral	4110000	GOLD
Donepezil 1mg/ml oral solution sugar free Donepezil hydrochloride 1mg/1ml Oral solution Oral	4110000	GOLD
Donepezil 5mg tablets (Waymade Healthcare Plc) Donepezil hydrochloride 5mg Tablet Oral	4110000	GOLD
Donepezil 10mg orodispersible tablets Donepezil hydrochloride 10mg Orodispersible tablet Oral	4110000	GOLD
Donepezil 10mg orodispersible tablets (DE Pharmaceuticals) Donepezil hydrochloride 10mg Orodispersible tablet Oral	4110000	GOLD
Donepezil 5mg tablets (Zentiva) Donepezil hydrochloride 5mg Tablet Oral	4110000	GOLD
Aricept Evess 10mg orodispersible tablets (Eisai Ltd) Donepezil hydrochloride 10mg Orodispersible tablet Oral	4110000	GOLD
Donepezil 10mg tablets (Teva UK Ltd) Donepezil hydrochloride 10mg Tablet Oral	4110000	GOLD
Donepezil 10mg tablets Donepezil hydrochloride 10mg Tablet Oral	4110000	GOLD
Donepezil 5mg tablets (A A H Pharmaceuticals Ltd) Donepezil hydrochloride 5mg Tablet Oral	4110000	GOLD
Donepezil 5mg tablets (Accord Healthcare Ltd) Donepezil hydrochloride 5mg Tablet Oral	4110000	GOLD
Donepezil 10mg orodispersible tablets sugar free Donepezil hydrochloride 10mg Orodispersible tablet Oral	4110000	GOLD
Aricept Evess 5mg orodispersible tablets (Eisai Ltd) Donepezil hydrochloride 5mg Orodispersible tablet Oral	4110000	GOLD
Donepezil 5mg tablets (Mylan) Donepezil hydrochloride 5mg Tablet Oral	4110000	GOLD
Aricept 5mg tablets (Waymade Healthcare Plc) Donepezil hydrochloride 5mg Tablet Oral	4110000	GOLD
Donepezil 10mg tablets (A A H Pharmaceuticals Ltd) Donepezil hydrochloride 10mg Tablet Oral	4110000	GOLD
Donepezil 5mg orodispersible tablets sugar free (A A H Pharmaceuticals Ltd) Donepezil hydrochloride 5mg Orodispersible tablet Oral	4110000	GOLD
Aricept 10mg tablets (Waymade Healthcare Plc) Donepezil hydrochloride 10mg Tablet Oral	4110000	GOLD
Donepezil 10mg tablets (Zentiva) Donepezil hydrochloride 10mg Tablet Oral	4110000	GOLD
Donepezil 10mg/5ml oral suspension Donepezil hydrochloride 2mg/1ml Oral suspension Oral	4110000	GOLD
Donepezil 5mg tablets Donepezil hydrochloride 5mg Tablet Oral	4110000	GOLD
Aricept 10mg tablets (Eisai Ltd) Donepezil hydrochloride 10mg Tablet Oral	4110000	GOLD
Aricept 5mg tablets (Eisai Ltd) Donepezil hydrochloride 5mg Tablet Oral	4110000	GOLD

© 2024 Wang J et al. *JAMA Network Open.*

Donepezil 10mg tablets (Mawdsley-Brooks & Company Ltd) Donepezil hydrochloride 10mg Tablet Oral	4110000	GOLD
Donepezil 10mg tablets (Accord Healthcare Ltd) Donepezil hydrochloride 10mg Tablet Oral	4110000	GOLD
Donepezil 10mg tablets (Phoenix Healthcare Distribution Ltd) Donepezil hydrochloride 10mg Tablet Oral	4110000	GOLD
Donepezil 5mg orodispersible tablets sugar free Donepezil hydrochloride 5mg Orodispersible tablet Oral	4110000	GOLD
Donepezil 10mg tablets (Actavis UK Ltd) Donepezil hydrochloride 10mg Tablet Oral	4110000	GOLD
Donepezil 5mg orodispersible tablets Donepezil hydrochloride 5mg Orodispersible tablet Oral	4110000	GOLD
Donepezil 5mg tablets (Ranbaxy (UK) Ltd)Donepezil hydrochloride5mgTabletOral	4110000	GOLD
Donepezil 5mg tablets (Pfizer Ltd)Donepezil hydrochloride5mgTabletOral	4110000	GOLD
Donepezil 10mg tablets (Pfizer Ltd)Donepezil hydrochloride10mgTabletOral	4110000	GOLD
Donepezil 5mg tablets Tablet Oral Donepezil hydrochloride 5.000mg	4110000	AURUM
Aricept 5mg tablets (Eisai Ltd) Tablet Oral Donepezil hydrochloride 5.000mg	4110000	AURUM
Aricept 10mg tablets (Eisai Ltd) Tablet Oral Donepezil hydrochloride 10.000mg	4110000	AURUM
Donepezil 10mg tablets Tablet Oral Donepezil hydrochloride 10.000mg	4110000	AURUM
Donepezil 10mg orodispersible tablets sugar free Orodispersible tablet Oral Donepezil hydrochloride 10.000mg	4110000	AURUM
Aricept Evess 10mg orodispersible tablets (Eisai Ltd) Orodispersible tablet Oral Donepezil hydrochloride 10.000mg	4110000	AURUM
Aricept Evess 5mg orodispersible tablets (Eisai Ltd) Orodispersible tablet Oral Donepezil hydrochloride 5.000mg	4110000	AURUM
Donepezil 5mg orodispersible tablets sugar free Orodispersible tablet Oral Donepezil hydrochloride 5.000mg	4110000	AURUM
Donepezil 1mg/ml oral solution sugar free Oral solution Oral Donepezil hydrochloride 1.000mg/1.000ml		AURUM

RIVASTIGMINE	BNF	DATABASE
Rivastigmine 4.6mg/24hours transdermal patches Rivastigmine 4.6mg/24hour Transdermal patch Transdermal	4110000	GOLD
Rivastigmine 3mg capsules (Dr Reddy's Laboratories (UK) Ltd) Rivastigmine hydrogen tartrate 3mg Capsule Oral	4110000	GOLD
Rivastigmine 3mg capsules Rivastigmine hydrogen tartrate 3mg Capsule Oral	4110000	GOLD
Exelon 1.5mg capsules (Novartis Pharmaceuticals UK Ltd) Rivastigmine hydrogen tartrate 1.5mg Capsule Oral	4110000	GOLD
Exelon 6mg capsules (Novartis Pharmaceuticals UK Ltd) Rivastigmine hydrogen tartrate 6mg Capsule Oral	4110000	GOLD
RIVASTIGMINE	0	GOLD
Prometax 9.5mg/24hours transdermal patches (Novartis Pharmaceuticals UK Ltd) Rivastigmine 9.5mg/24hour Transdermal patch Transdermal	4110000	GOLD
Rivastigmine 6mg capsules (Waymade Healthcare Plc) Rivastigmine hydrogen tartrate 6mg Capsule Oral	4110000	GOLD
Eluden 4.6mg/24hours transdermal patches (Mylan) Rivastigmine 4.6mg/24hour Transdermal patch Transdermal	4110000	GOLD

© 2024 Wang J et al. *JAMA Network Open.*

Almuriva 9.5mg/24hours transdermal patches (Sandoz Ltd) Rivastigmine 9.5mg/24hour Transdermal patch Transdermal	4110000	GOLD
RIVASTIGMINE	0	GOLD
Voleze 9.5mg/24hours transdermal patches (Focus Pharmaceuticals Ltd) Rivastigmine 9.5mg/24hour Transdermal patch Transdermal	4110000	GOLD
RIVASTIGMINE	0	GOLD
Rivastigmine 4.5mg capsules Rivastigmine hydrogen tartrate 4.5mg Capsule Oral	4110000	GOLD
Rivastigmine 6mg capsules (A A H Pharmaceuticals Ltd) Rivastigmine hydrogen tartrate 6mg Capsule Oral	4110000	GOLD
Prometax 4.6mg/24hours transdermal patches (Novartis Pharmaceuticals UK Ltd) Rivastigmine 4.6mg/24hour Transdermal patch Transdermal	4110000	GOLD
Rivastigmine 2mg/ml oral solution Rivastigmine hydrogen tartrate 2mg/1ml Oral solution Oral	4110000	GOLD
Rivastigmine 9.5mg/24hours transdermal patches (DE Pharmaceuticals) Rivastigmine 9.5mg/24hour Transdermal patch Transdermal	4110000	GOLD
Exelon 4.5mg capsules (Waymade Healthcare Plc) Rivastigmine hydrogen tartrate 4.5mg Capsule Oral	4110000	GOLD
Eluden 9.5mg/24hours transdermal patches (Mylan) Rivastigmine 9.5mg/24hour Transdermal patch Transdermal	4110000	GOLD
Alzest 9.5mg/24hours transdermal patches (Dr Reddy's Laboratories (UK) Ltd) Rivastigmine 9.5mg/24hour Transdermal patch Transdermal	4110000	GOLD
Rivastigmine 13.3mg/24hours transdermal patches Rivastigmine 13.3mg/24hour Transdermal patch Transdermal	4110000	GOLD
Alzest 4.6mg/24hours transdermal patches (Dr Reddy's Laboratories (UK) Ltd) Rivastigmine 4.6mg/24hour Transdermal patch Transdermal	4110000	GOLD
Voleze 4.6mg/24hours transdermal patches (Advanz Pharma) Rivastigmine 4.6mg/24hour Transdermal patch Transdermal	4110000	GOLD
Exelon 9.5mg/24hours transdermal patches (Novartis Pharmaceuticals UK Ltd) Rivastigmine 9.5mg/24hour Transdermal patch Transdermal	4110000	GOLD
Rivastigmine 9.5mg/24hours transdermal patches (Actavis UK Ltd) Rivastigmine 9.5mg/24hour Transdermal patch Transdermal	4110000	GOLD
Almuriva 4.6mg/24hours transdermal patches (Sandoz Ltd) Rivastigmine 4.6mg/24hour Transdermal patch Transdermal	4110000	GOLD
Erastig 4.6mg/24hours transdermal patches (Teva UK Ltd) Rivastigmine 4.6mg/24hour Transdermal patch Transdermal	4110000	GOLD
Rivastigmine 2mg/ml oral solution sugar free Rivastigmine hydrogen tartrate 2mg/1ml Oral solution Oral	4110000	GOLD
Exelon 4.6mg/24hours transdermal patches (Novartis Pharmaceuticals UK Ltd) Rivastigmine 4.6mg/24hour Transdermal patch Transdermal	4110000	GOLD
Rivastigmine 9.5mg/24hours transdermal patches (A A H Pharmaceuticals Ltd) Rivastigmine 9.5mg/24hour Transdermal patch Transdermal	4110000	GOLD
Rivastigmine 4.6mg/24hours transdermal patches (A A H Pharmaceuticals Ltd) Rivastigmine 4.6mg/24hour Transdermal patch Transdermal	4110000	GOLD
Exelon 3mg capsules (Novartis Pharmaceuticals UK Ltd) Rivastigmine hydrogen tartrate 3mg Capsule Oral	4110000	GOLD
Exelon 13.3mg/24hours transdermal patches (Novartis Pharmaceuticals UK Ltd) Rivastigmine 13.3mg/24hour Transdermal patch Transdermal	4110000	GOLD
Rivastigmine 1.5mg capsules Rivastigmine hydrogen tartrate 1.5mg Capsule Oral	4110000	GOLD
Erastig 9.5mg/24hours transdermal patches (Teva UK Ltd) Rivastigmine 9.5mg/24hour Transdermal patch Transdermal	4110000	GOLD

© 2024 Wang J et al. *JAMA Network Open.*

Rivastigmine 6mg capsules Rivastigmine hydrogen tartrate 6mg Capsule Oral	4110000	GOLD
Exelon 4.5mg capsules (Novartis Pharmaceuticals UK Ltd) Rivastigmine hydrogen tartrate 4.5mg Capsule Oral	4110000	GOLD
Rivastigmine 4.6mg/24hours transdermal patches (Actavis UK Ltd) Rivastigmine 4.6mg/24hour Transdermal patch Transdermal	4110000	GOLD
Rivastigmine 9.5mg/24hours transdermal patches Rivastigmine 9.5mg/24hour Transdermal patch Transdermal	4110000	GOLD
Exelon 2mg/ml oral solution (Novartis Pharmaceuticals UK Ltd) Rivastigmine hydrogen tartrate 2mg/1ml Oral solution Oral	4110000	GOLD
Rivastigmine 1.5mg capsules Capsule Oral Rivastigmine hydrogen tartrate 1.500mg	4110000	AURUM
Exelon 1.5mg capsules (Novartis Pharmaceuticals UK Ltd) Capsule Oral Rivastigmine hydrogen tartrate 1.500mg	4110000	AURUM
Nimvastid 1.5mg capsules (Consilient Health Ltd) Capsule Oral Rivastigmine hydrogen tartrate 1.500mg		AURUM
Kerstipon 1.5mg capsules (Aspire Pharma Ltd) Capsule Oral Rivastigmine hydrogen tartrate 1.500mg		AURUM
Kerstipon 3mg capsules (Aspire Pharma Ltd) Capsule Oral Rivastigmine hydrogen tartrate 3.000mg		AURUM
Nimvastid 3mg capsules (Consilient Health Ltd) Capsule Oral Rivastigmine hydrogen tartrate 3.000mg		AURUM
Exelon 3mg capsules (Novartis Pharmaceuticals UK Ltd) Capsule Oral Rivastigmine hydrogen tartrate 3.000mg	4110000	AURUM
Rivastigmine 3mg capsules Capsule Oral Rivastigmine hydrogen tartrate 3.000mg	4110000	AURUM
Rivastigmine 4.5mg capsules Capsule Oral Rivastigmine hydrogen tartrate 4.500mg	4110000	AURUM
Exelon 4.5mg capsules (Novartis Pharmaceuticals UK Ltd) Capsule Oral Rivastigmine hydrogen tartrate 4.500mg	4110000	AURUM
Nimvastid 4.5mg capsules (Consilient Health Ltd) Capsule Oral Rivastigmine hydrogen tartrate 4.500mg		AURUM
Kerstipon 4.5mg capsules (Aspire Pharma Ltd) Capsule Oral Rivastigmine hydrogen tartrate 4.500mg		AURUM
Kerstipon 6mg capsules (Aspire Pharma Ltd) Capsule Oral Rivastigmine hydrogen tartrate 6.000mg		AURUM
Nimvastid 6mg capsules (Consilient Health Ltd) Capsule Oral Rivastigmine hydrogen tartrate 6.000mg		AURUM
Exelon 6mg capsules (Novartis Pharmaceuticals UK Ltd) Capsule Oral Rivastigmine hydrogen tartrate 6.000mg	4110000	AURUM
Rivastigmine 6mg capsules Capsule Oral Rivastigmine hydrogen tartrate 6.000mg	4110000	AURUM
Rivastigmine 2mg/ml oral solution sugar free Oral solution Oral Rivastigmine hydrogen tartrate 2.000mg/1.000ml	4110000	AURUM
Exelon 2mg/ml oral solution (Novartis Pharmaceuticals UK Ltd) Oral solution Oral Rivastigmine hydrogen tartrate 2.000mg/1.000ml	4110000	AURUM
Rivastigmine 4.6mg/24hours transdermal patches Transdermal patch Transdermal Rivastigmine 4.600mg/24.000hour	4110000	AURUM
Exelon 4.6mg/24hours transdermal patches (Novartis Pharmaceuticals UK Ltd) Transdermal patch Transdermal Rivastigmine 4.600mg/24.000hour	4110000	AURUM

© 2024 Wang J et al. *JAMA Network Open.*

Erastig 4.6mg/24hours transdermal patches (Teva UK Ltd) Transdermal patch Transdermal Rivastigmine 4.600mg/24.000hour		AURUM
Voleze 4.6mg/24hours transdermal patches (Advanz Pharma) Transdermal patch Transdermal Rivastigmine 4.600mg/24.000hour		AURUM
Almuriva 4.6mg/24hours transdermal patches (Sandoz Ltd) Transdermal patch Transdermal Rivastigmine 4.600mg/24.000hour		AURUM
Prometax 4.6mg/24hours transdermal patches (Novartis Pharmaceuticals UK Ltd) Transdermal patch Transdermal Rivastigmine 4.600mg/24.000hour		AURUM
Somniton 4.6mg/24hours transdermal patches (Zentiva) Transdermal patch Transdermal Rivastigmine 4.600mg/24.000hour		AURUM
Eluden 4.6mg/24hours transdermal patches (Mylan) Transdermal patch Transdermal Rivastigmine 4.600mg/24.000hour		AURUM
Alzest 4.6mg/24hours transdermal patches (Dr Reddy's Laboratories (UK) Ltd) Transdermal patch Transdermal Rivastigmine 4.600mg/24.000hour		AURUM
Voleze 9.5mg/24hours transdermal patches (Focus Pharmaceuticals Ltd) Transdermal patch Transdermal Rivastigmine 9.500mg/24.000hour		AURUM
Erastig 9.5mg/24hours transdermal patches (Teva UK Ltd) Transdermal patch Transdermal Rivastigmine 9.500mg/24.000hour		AURUM
Exelon 9.5mg/24hours transdermal patches (Novartis Pharmaceuticals UK Ltd) Transdermal patch Transdermal Rivastigmine 9.500mg/24.000hour	4110000	AURUM
Rivastigmine 9.5mg/24hours transdermal patches Transdermal patch Transdermal Rivastigmine 9.500mg/24.000hour	4110000	AURUM
Alzest 9.5mg/24hours transdermal patches (Dr Reddy's Laboratories (UK) Ltd) Transdermal patch Transdermal Rivastigmine 9.500mg/24.000hour		AURUM
Eluden 9.5mg/24hours transdermal patches (Mylan) Transdermal patch Transdermal Rivastigmine 9.500mg/24.000hour		AURUM
Prometax 9.5mg/24hours transdermal patches (Novartis Pharmaceuticals UK Ltd) Transdermal patch Transdermal Rivastigmine 9.500mg/24.000hour		AURUM
Somniton 9.5mg/24hours transdermal patches (Zentiva) Transdermal patch Transdermal Rivastigmine 9.500mg/24.000hour		AURUM
Almuriva 9.5mg/24hours transdermal patches (Sandoz Ltd) Transdermal patch Transdermal Rivastigmine 9.500mg/24.000hour		AURUM
Exelon 13.3mg/24hours transdermal patches (Novartis Pharmaceuticals UK Ltd) Transdermal patch Transdermal Rivastigmine 13.300mg/24.000hour		AURUM
Rivastigmine 13.3mg/24hours transdermal patches Transdermal patch Transdermal Rivastigmine 13.300mg/24.000hour		AURUM
Erastig 13.3mg/24hours transdermal patches (Teva UK Ltd) Transdermal patch Transdermal Rivastigmine 13.300mg/24.000hour		AURUM
Voleze 13.3mg/24hours transdermal patches (Advanz Pharma) Transdermal patch Transdermal Rivastigmine 13.300mg/24.000hour		AURUM

GALANTAMINE	BNF	DATABASE
Lotprosin XL 16mg capsules (Accord Healthcare Ltd) Galantamine hydrobromide 16mg Modified-release capsule Oral	4110000	GOLD
Galantamine 20mg/5ml oral solution sugar free Galantamine hydrobromide 4mg/1ml Oral solution Oral	4110000	GOLD
Reminyl 12mg tablets (Shire Pharmaceuticals Ltd) Galantamine hydrobromide 12mg Tablet Oral	4110000	GOLD

© 2024 Wang J et al. *JAMA Network Open.*

Zeebral XL 16mg capsules (Teva UK Ltd) Galantamine hydrobromide 16mg Modified-release capsule Oral	4110000	GOLD
Galzemic 4mg/ml oral solution (Creo Pharma Ltd) Galantamine hydrobromide 4mg/1ml Oral solution Oral	4110000	GOLD
Galantamine 8mg modified-release capsules Galantamine hydrobromide 8mg Modified-release capsule Oral	4110000	GOLD
Reminyl XL 16mg capsules (Shire Pharmaceuticals Ltd) Galantamine hydrobromide 16mg Modified-release capsule Oral	4110000	GOLD
Acumor XL 24mg capsules (Mylan) Galantamine hydrobromide 24mg Modified-release capsule Oral	4110000	GOLD
Reminyl XL 24mg capsules (Shire Pharmaceuticals Ltd) Galantamine hydrobromide 24mg Modified-release capsule Oral	4110000	GOLD
Galantamine 12mg tablets Galantamine hydrobromide 12mg Tablet Oral	4110000	GOLD
Reminyl 4mg tablets (Shire Pharmaceuticals Ltd) Galantamine hydrobromide 4mg Tablet Oral	4110000	GOLD
Galzemic XL 16mg capsules (Creo Pharma Ltd) Galantamine hydrobromide 16mg Modified-release capsule Oral	4110000	GOLD
Acumor XL 16mg capsules (Mylan) Galantamine hydrobromide 16mg Modified-release capsule Oral	4110000	GOLD
Galantamine 8mg tablets Galantamine hydrobromide 8mg Tablet Oral	4110000	GOLD
Galsya XL 16mg capsules (Consilient Health Ltd) Galantamine hydrobromide 16mg Modified-release capsule Oral	4110000	GOLD
Luventa XL 8mg capsules (Fontus Health Ltd) Galantamine hydrobromide 8mg Modified-release capsule Oral	4110000	GOLD
Galsya XL 8mg capsules (Consilient Health Ltd) Galantamine hydrobromide 8mg Modified-release capsule Oral	4110000	GOLD
Gatalin XL 24mg capsules (Aspire Pharma Ltd) Galantamine hydrobromide 24mg Modified-release capsule Oral	4110000	GOLD
Galzemic XL 8mg capsules (Creo Pharma Ltd) Galantamine hydrobromide 8mg Modified-release capsule Oral	4110000	GOLD
Reminyl 4mg/ml oral solution (Shire Pharmaceuticals Ltd) Galantamine hydrobromide 4mg/1ml Oral solution Oral	4110000	GOLD
Gazylan XL 16mg capsules (Teva UK Ltd) Galantamine hydrobromide 16mg Modified-release capsule Oral	4110000	GOLD
Luventa XL 24mg capsules (Fontus Health Ltd) Galantamine hydrobromide 24mg Modified-release capsule Oral	4110000	GOLD
Gazylan XL 24mg capsules (Teva UK Ltd) Galantamine hydrobromide 24mg Modified-release capsule Oral	4110000	GOLD
Galantamine 4mg tablets Galantamine hydrobromide 4mg Tablet Oral	4110000	GOLD
Gazylan XL 8mg capsules (Teva UK Ltd) Galantamine hydrobromide 8mg Modified-release capsule Oral	4110000	GOLD
Galzemic XL 24mg capsules (Creo Pharma Ltd) Galantamine hydrobromide 24mg Modified-release capsule Oral	4110000	GOLD
Reminyl XL 8mg capsules (Shire Pharmaceuticals Ltd) Galantamine hydrobromide 8mg Modified-release capsule Oral	4110000	GOLD
Gatalin XL 16mg capsules (Aspire Pharma Ltd) Galantamine hydrobromide 16mg Modified-release capsule Oral	4110000	GOLD
Galantamine 24mg modified-release capsules Galantamine hydrobromide 24mg Modified-release capsule Oral	4110000	GOLD

© 2024 Wang J et al. *JAMA Network Open.*

Gatalin XL 8mg capsules (Aspire Pharma Ltd) Galantamine hydrobromide 8mg Modified-release capsule Oral	4110000	GOLD
Luventa XL 16mg capsules (Fontus Health Ltd) Galantamine hydrobromide 16mg Modified-release capsule Oral	4110000	GOLD
Galantamine 16mg modified-release capsules Galantamine hydrobromide 16mg Modified-release capsule Oral	4110000	GOLD
Galsya XL 24mg capsules (Consilient Health Ltd) Galantamine hydrobromide 24mg Modified-release capsule Oral	4110000	GOLD
Reminyl 8mg tablets (Shire Pharmaceuticals Ltd) Galantamine hydrobromide 8mg Tablet Oral	4110000	GOLD
Galantamine 8mg tablets (A A H Pharmaceuticals Ltd) Galantamine hydrobromide 8mg Tablet Oral	4110000	GOLD
Galantamine 4mg tablets Tablet Oral Galantamine hydrobromide 4.000mg	4110000	AURUM
Reminyl 4mg tablets (Shire Pharmaceuticals Ltd) Tablet Oral Galantamine hydrobromide 4.000mg	4110000	AURUM
Reminyl 8mg tablets (Shire Pharmaceuticals Ltd) Tablet Oral Galantamine hydrobromide 8.000mg	4110000	AURUM
Galantamine 8mg tablets Tablet Oral Galantamine hydrobromide 8.000mg	4110000	AURUM
Galantamine 12mg tablets Tablet Oral Galantamine hydrobromide 12.000mg	4110000	AURUM
Reminyl 12mg tablets (Shire Pharmaceuticals Ltd) Tablet Oral Galantamine hydrobromide 12.000mg	4110000	AURUM
Galantamine 20mg/5ml oral solution sugar free Oral solution Oral Galantamine hydrobromide 4.000mg/1.000ml	4110000	AURUM
Reminyl 4mg/ml oral solution (Shire Pharmaceuticals Ltd) Oral solution Oral Galantamine hydrobromide 4.000mg/1.000ml	4110000	AURUM
Galzemic 4mg/ml oral solution (Creo Pharma Ltd) Oral solution Oral Galantamine hydrobromide 4.000mg/1.000ml		AURUM
Galsya XL 16mg capsules (Consilient Health Ltd) Modified-release capsule Oral Galantamine hydrobromide 16.000mg	4110000	AURUM
Zeebral XL 16mg capsules (Teva UK Ltd) Modified-release capsule Oral Galantamine hydrobromide 16.000mg		AURUM
Gatalin XL 16mg capsules (Aspire Pharma Ltd) Modified-release capsule Oral Galantamine hydrobromide 16.000mg		AURUM
Lotprosin XL 16mg capsules (Accord Healthcare Ltd) Modified-release capsule Oral Galantamine hydrobromide 16.000mg		AURUM
Acumor XL 16mg capsules (Mylan) Modified-release capsule Oral Galantamine hydrobromide 16.000mg		AURUM
Elmino XL 16mg capsules (Zentiva) Modified-release capsule Oral Galantamine hydrobromide 16.000mg		AURUM
Galantamine 16mg modified-release capsules Modified-release capsule Oral Galantamine hydrobromide 16.000mg	4110000	AURUM
Reminyl XL 16mg capsules (Shire Pharmaceuticals Ltd) Modified-release capsule Oral Galantamine hydrobromide 16.000mg	4110000	AURUM
Gaalin 16mg modified-release capsules (Milpharm Ltd) Modified-release capsule Oral Galantamine hydrobromide 16.000mg		AURUM
Luventa XL 16mg capsules (Fontus Health Ltd) Modified-release capsule Oral Galantamine hydrobromide 16.000mg		AURUM
Galzemic XL 16mg capsules (Creo Pharma Ltd) Modified-release capsule Oral Galantamine hydrobromide 16.000mg		AURUM

© 2024 Wang J et al. *JAMA Network Open.*

Consion XL 16mg capsules (Dr Reddy's Laboratories (UK) Ltd) Modified-release capsule Oral Galantamine hydrobromide 16.000mg		AURUM
Gazylan XL 16mg capsules (Teva UK Ltd) Modified-release capsule Oral Galantamine hydrobromide 16.000mg		AURUM
Galantamine 24mg modified-release capsules Modified-release capsule Oral Galantamine hydrobromide 24.000mg	4110000	AURUM
Reminyl XL 24mg capsules (Shire Pharmaceuticals Ltd) Modified-release capsule Oral Galantamine hydrobromide 24.000mg	4110000	AURUM
Elmino XL 24mg capsules (Zentiva) Modified-release capsule Oral Galantamine hydrobromide 24.000mg		AURUM
Acumor XL 24mg capsules (Mylan) Modified-release capsule Oral Galantamine hydrobromide 24.000mg		AURUM
Lotprosin XL 24mg capsules (Accord Healthcare Ltd) Modified-release capsule Oral Galantamine hydrobromide 24.000mg		AURUM
Gatalin XL 24mg capsules (Aspire Pharma Ltd) Modified-release capsule Oral Galantamine hydrobromide 24.000mg		AURUM
Zeebral XL 24mg capsules (Teva UK Ltd) Modified-release capsule Oral Galantamine hydrobromide 24.000mg		AURUM
Galsya XL 24mg capsules (Consilient Health Ltd) Modified-release capsule Oral Galantamine hydrobromide 24.000mg	4110000	AURUM
Gazylan XL 24mg capsules (Teva UK Ltd) Modified-release capsule Oral Galantamine hydrobromide 24.000mg		AURUM
Consion XL 24mg capsules (Dr Reddy's Laboratories (UK) Ltd) Modified-release capsule Oral Galantamine hydrobromide 24.000mg		AURUM
Galzemic XL 24mg capsules (Creo Pharma Ltd) Modified-release capsule Oral Galantamine hydrobromide 24.000mg		AURUM
Luventa XL 24mg capsules (Fontus Health Ltd) Modified-release capsule Oral Galantamine hydrobromide 24.000mg		AURUM
Gaalin 24mg modified-release capsules (Milpharm Ltd) Modified-release capsule Oral Galantamine hydrobromide 24.000mg		AURUM
Galsya XL 8mg capsules (Consilient Health Ltd) Modified-release capsule Oral Galantamine hydrobromide 8.000mg	4110000	AURUM
Zeebral XL 8mg capsules (Teva UK Ltd) Modified-release capsule Oral Galantamine hydrobromide 8.000mg		AURUM
Gatalin XL 8mg capsules (Aspire Pharma Ltd) Modified-release capsule Oral Galantamine hydrobromide 8.000mg		AURUM
Lotprosin XL 8mg capsules (Accord Healthcare Ltd) Modified-release capsule Oral Galantamine hydrobromide 8.000mg		AURUM
Acumor XL 8mg capsules (Mylan) Modified-release capsule Oral Galantamine hydrobromide 8.000mg		AURUM
Elmino XL 8mg capsules (Zentiva) Modified-release capsule Oral Galantamine hydrobromide 8.000mg		AURUM
Galantamine 8mg modified-release capsules Modified-release capsule Oral Galantamine hydrobromide 8.000mg	4110000	AURUM
Reminyl XL 8mg capsules (Shire Pharmaceuticals Ltd) Modified-release capsule Oral Galantamine hydrobromide 8.000mg	4110000	AURUM
Gaalin 8mg modified-release capsules (Milpharm Ltd) Modified-release capsule Oral Galantamine hydrobromide 8.000mg		AURUM
Luventa XL 8mg capsules (Fontus Health Ltd) Modified-release capsule Oral Galantamine hydrobromide 8.000mg		AURUM

© 2024 Wang J et al. *JAMA Network Open.*

Galzemic XL 8mg capsules (Creo Pharma Ltd) Modified-release capsule Oral Galantamine hydrobromide 8.000mg		AURUM
Consion XL 8mg capsules (Dr Reddy's Laboratories (UK) Ltd) Modified-release capsule Oral Galantamine hydrobromide 8.000mg		AURUM
Gazylan XL 8mg capsules (Teva UK Ltd) Modified-release capsule Oral Galantamine hydrobromide 8.000mg		AURUM

MEMANTINE	BNF	DATABASE
Ebixa 5mg/0.5ml pump actuation oral solution (Lundbeck Ltd) Memantine hydrochloride 10mg/1ml Oral solution Oral	4110000	GOLD
Memantine 5mg tablets Memantine hydrochloride 5mg Tablet Oral	4110000	GOLD
Memantine 5mg/10mg/15mg/20mg tablets treatment initiation pack Not applicable Route of administration not applicable	4110000	GOLD
Memantine 10mg/ml oral solution sugar free (A A H Pharmaceuticals Ltd) Memantine hydrochloride 10mg/1ml Oral solution Oral	4110000	GOLD
Memantine 5mg+10mg+15mg+20mg Tablet Memantine Hydrochloride 5mg+10mg+15mg+20mg Tablet Oral	4110000	GOLD
Memantine 10mg/ml oral solution sugar free (Healthcare Pharma Ltd) Memantine hydrochloride 10mg/1ml Oral solution Oral	4110000	GOLD
Nemdatine 10mg tablets (Actavis UK Ltd) Memantine hydrochloride 10mg Tablet Oral	4110000	GOLD
Memantine 10mg tablets (A A H Pharmaceuticals Ltd) Memantine hydrochloride 10mg Tablet Oral	4110000	GOLD
Memantine 10mg orodispersible tablets sugar free Memantine hydrochloride 10mg Orodispersible tablet Oral	4110000	GOLD
Memantine 5mg/10mg/15mg/20mg tablets treatment initiation pack (Lupin Healthcare (UK) Ltd) Not applicable Route of administration not applicable	4110000	GOLD
Ebixa 20mg tablets (Lundbeck Ltd) Memantine hydrochloride 20mg Tablet Oral	4110000	GOLD
Memantine 20mg orodispersible tablets sugar free Memantine hydrochloride 20mg Orodispersible tablet Oral	4110000	GOLD
Ebixa tablets treatment initiation pack (Lundbeck Ltd) Memantine Hydrochloride 5mg+10mg+15mg+20mg Not applicable Route of administration not applicable	4110000	GOLD
Memantine 10mg/ml oral solution sugar free (Alliance Healthcare (Distribution) Ltd) Memantine hydrochloride 10mg/1ml Oral solution Oral	4110000	GOLD
Memantine 20mg tablets Memantine hydrochloride 20mg Tablet Oral	4110000	GOLD
Memantine 10mg/ml oral solution sugar free (Teva UK Ltd) Memantine hydrochloride 10mg/1ml Oral solution Oral	4110000	GOLD
Memantine 5mg/10mg/15mg/20mg orodispersible tablets initiation pack sugar free Not applicable Route of administration not applicable	4110000	GOLD
Ebixa 10mg tablets (DE Pharmaceuticals) Memantine hydrochloride 10mg Tablet Oral	4110000	GOLD
Nemdatine 20mg tablets (Actavis UK Ltd) Memantine hydrochloride 20mg Tablet Oral	4110000	GOLD
Memantine 10mg/ml oral solution sugar free Memantine hydrochloride 10mg/1ml Oral solution Oral	4110000	GOLD
Memantine 10mg soluble tablets sugar free Memantine hydrochloride 10mg Soluble tablet Oral	4110000	GOLD

© 2024 Wang J et al. *JAMA Network Open.*

Memantine 20mg soluble tablets sugar free Memantine hydrochloride 20mg Soluble tablet Oral	4110000	GOLD
Memantine 10mg/ml oral solution sugar free (Zentiva) Memantine hydrochloride 10mg/1ml Oral solution Oral	4110000	GOLD
Marixino 20mg tablets (Consilient Health Ltd) Memantine hydrochloride 20mg Tablet Oral	4110000	GOLD
Ebixa 10mg tablets (Lundbeck Ltd) Memantine hydrochloride 10mg Tablet Oral	4110000	GOLD
Memantine 10mg tablets Memantine hydrochloride 10mg Tablet Oral	4110000	GOLD
Memantine 5mg/10mg/15mg/20mg tablets treatment initiation pack (A A H Pharmaceuticals Ltd) Not applicable Route of administration not applicable	4110000	GOLD
Memantine 20mg tablets (Teva UK Ltd) Memantine hydrochloride 20mg Tablet Oral	4110000	GOLD
Valios 20mg orodispersible tablets sugar free (Dr Reddy's Laboratories (UK) Ltd)Memantine hydrochloride20mgOrodispersible tabletOral	4110000	GOLD
Memantine 5mg/10mg/15mg/20mg tablets treatment initiation pack (Torrent Pharma (UK) Ltd)Not applicableRoute of administration not applicable	4110000	GOLD
Memantine 10mg tablets (Niche Generics Ltd)Memantine hydrochloride10mgTabletOral	4110000	GOLD
Memantine 20mg tablets (Niche Generics Ltd)Memantine hydrochloride20mgTabletOral	4110000	GOLD
Memantine 10mg tablets Tablet Oral Memantine hydrochloride 10.000mg	4110000	AURUM
Ebixa 10mg tablets (Lundbeck Ltd) Tablet Oral Memantine hydrochloride 10.000mg	4110000	AURUM
Marixino 10mg tablets (Consilient Health Ltd) Tablet Oral Memantine hydrochloride 10.000mg		AURUM
Nemdatine 10mg tablets (Actavis UK Ltd) Tablet Oral Memantine hydrochloride 10.000mg		AURUM
Memantine 10mg/ml oral solution sugar free Oral solution Oral Memantine hydrochloride 10.000mg/1.000ml	4110000	AURUM
Ebixa 5mg/0.5ml pump actuation oral solution (Lundbeck Ltd) Oral solution Oral Memantine hydrochloride 10.000mg/1.000ml	4110000	AURUM
Memantine 10mg soluble tablets sugar free Soluble tablet Oral Memantine hydrochloride 10.000mg		AURUM
Alzhok 10mg soluble tablets (Glenmark Pharmaceuticals Europe Ltd) Soluble tablet Oral Memantine hydrochloride 10.000mg		AURUM
Memantine 20mg soluble tablets sugar free Soluble tablet Oral Memantine hydrochloride 20.000mg		AURUM
Alzhok 20mg soluble tablets (Glenmark Pharmaceuticals Europe Ltd) Soluble tablet Oral Memantine hydrochloride 20.000mg		AURUM
Memantine 20mg tablets Tablet Oral Memantine hydrochloride 20.000mg		AURUM
Ebixa 20mg tablets (Lundbeck Ltd) Tablet Oral Memantine hydrochloride 20.000mg		AURUM
Marixino 20mg tablets (Consilient Health Ltd) Tablet Oral Memantine hydrochloride 20.000mg		AURUM
Nemdatine 20mg tablets (Actavis UK Ltd) Tablet Oral Memantine hydrochloride 20.000mg		AURUM
Memantine 5mg/10mg/15mg/20mg tablets treatment initiation pack Not applicable Route of administration not applicable		AURUM
Ebixa tablets treatment initiation pack (Lundbeck Ltd) Not applicable		AURUM

© 2024 Wang J et al. *JAMA Network Open.*

Memantine 10mg orodispersible tablets sugar free Orodispersible tablet Oral Memantine hydrochloride 10.000mg	4110000	AURUM
Valios 10mg orodispersible tablets sugar free (Dr Reddy's Laboratories (UK) Ltd) Orodispersible tablet Oral Memantine hydrochloride 10.000mg	4110000	AURUM
Valios 20mg orodispersible tablets sugar free (Dr Reddy's Laboratories (UK) Ltd) Orodispersible tablet Oral Memantine hydrochloride 20.000mg	4110000	AURUM
Memantine 20mg orodispersible tablets sugar free Orodispersible tablet Oral Memantine hydrochloride 20.000mg	4110000	AURUM
Valios 5mg/10mg/15mg/20mg orodispersible tablets initiation pack (Dr Reddy's Laboratories (UK) Ltd) Not applicable		AURUM
Memantine 5mg/10mg/15mg/20mg orodispersible tablets initiation pack sugar free Not applicable Route of administration not applicable		AURUM

© 2024 Wang J et al. *JAMA Network Open.*

eTable 3. Clinical Codes for Age-Related Macular Degeneration

DESCRIPTION	READ CODE	SNOMED-CT CODE	DATABASE
Unspecified senile macular degeneration	F425000		GOLD
Drusen of optic disc	F4H2000		GOLD
Drusen	F425700		GOLD
Senile macular degeneration	F425.11		GOLD
Retinal drusen	2BBH.00		GOLD
Dry senile macular degeneration	F425100		GOLD
Wet senile macular degeneration	F425200		GOLD
Senile reticular degeneration	F426400		GOLD
Kuhnt - Junius degeneration	F425211		GOLD
Drusen of optic disc	F4H2000	33629003	AURUM
Retinal drusen	2BBH.00	247153005	AURUM
Senile macular degeneration	F425.11	267718000	AURUM
Senile reticular degeneration	F426400	54184008	AURUM
Drusen	F425700	18695008	AURUM
Unspecified senile macular degeneration	F425000	267718000	AURUM
Optic nerve head drusen		33629003	AURUM
Nonneovascular age-related macular degeneration		414875008	AURUM
Nonexudative senile macular retinal degeneration		414875008	AURUM
Atrophic senile macular retinal degeneration		414875008	AURUM
Optic disc drusen		33629003	AURUM
Atrophic age-related macular degeneration		414875008	AURUM
Exudative senile macular retinal degeneration		414173003	AURUM
AMD - Age-related macular degeneration		267718000	AURUM
Nonexudative age-related macular degeneration		414875008	AURUM
ARMD - Age-related macular degeneration		267718000	AURUM
Neovascular age-related macular degeneration		414173003	AURUM
AAMD - Age related macular degeneration		267718000	AURUM
Dry senile macular retinal degeneration		414875008	AURUM
Senile reticular retinal degeneration		54184008	AURUM
SMD - Senile macular degeneration		267718000	AURUM
Dry senile macular degeneration	F425100	414875008	AURUM
Wet senile macular degeneration	F425200	414173003	AURUM
Kuhnt - Junius degeneration	F425211	414173003	AURUM
Age-related macular degeneration		267718000	AURUM
Kuhnt Junius degeneration		414173003	AURUM
Geographic atrophy of the macula		414875008	AURUM
Exudative age-related macular degeneration		414173003	AURUM
Nonexudative senile macular degeneration		414875008	AURUM
Junius-Kuhnt degeneration		414173003	AURUM
Disciform senile macular retinal degeneration		414173003	AURUM
EMD - Exudative macular degeneration		414173003	AURUM

© 2024 Wang J et al. *JAMA Network Open.*

© 2024 Wang J et al. *JAMA Network Open.*

eTable 4. Baseline Demographic Characteristics, Behavioural Risk Factors, Dementia Related Characteristics, Comorbidities, and Metabolic Characteristics of Unweighted Cohort One

	**Unweighted Cohort One**	
	**Donepezil (n = 104,237)**	**Rivastigmine/ Galantamine (n = 28,609)**
**Sociodemographic characteristics**		
Mean age, years (SD)	80.4 (7.7)	80.6 (7.4)
Male sex, n (%)	38308 (36.8)	12406 (43.4)
Ethnicity, n (%)		
White	57711 (55.4)	14577 (51.0)
South Asian	1574 (1.5)	368 (1.3)
Black	2293 (2.2)	495 (1.7)
Mixed Race	193 (0.2)	39 (0.1)
Others	575 (0.6)	153 (0.5)
Missing	41891 (40.2)	12977 (45.4)
**Behavioural/lifestyle risk factors**		
Mean BMI kg/m^2 (SD)	25.5 (4.8)	25.3 (4.9)
Smoking status, n (%)		
Non-smokers	40811 (39.2)	11223 (39.2)
Ex-smokers	44426 (42.6)	12417 (43.4)
Current smokers	16743 (16.1)	4335 (15.2)
Missing	2257 (2.2)	634 (2.2)
Drinking status, n (%)		
Non-drinkers	10974 (10.5)	3263 (11.4)
Ex-drinkers	5503 (5.3)	1627 (5.7)
Current drinkers	80043 (76.8)	21376 (74.7)
Missing	7717 (7.4)	2343 (8.2)
**Dementia-related characteristics**		
Median dementia duration in years [IQR]	0.3 [0.1, 0.7]	1.4 [0.8, 2.1]
Vascular dementia, n (%)	8342 (8.0)	2964 (10.4)
Other dementia, n (%)	61141 (58.7)	24124 (84.3)
Alzheimer's disease, n (%)	72667 (69.7)	14743 (51.5)
**Comorbidities**, n (%)		
Hypertension	56840 (54.5)	14916 (52.1)
Chronic kidney disease	23269 (22.3)	6753 (23.6)
Ischemic heart disease	18622 (17.9)	5972 (20.9)
Stroke/TIA	12373 (11.9)	4135 (14.5)
Myocardial infarction	6234 (6.0)	2173 (7.6)
Heart failure	5633 (5.4)	1873 (6.5)

© 2024 Wang J et al. *JAMA Network Open.*

Atrial fibrillation	10573 (10.1)	3411 (11.9)
Peripheral vascular disease	2624 (2.5)	761 (2.7)
Aortic atheroma & plaque	3763 (3.6)	1227 (4.3)
Type 1 diabetes	655 (0.6)	184 (0.6)
Type 2 diabetes	15850 (15.2)	4293 (15.0)
Peripheral Neuropathy	8042 (7.7)	1981 (6.9)
Diabetic retinopathy	5454 (5.3)	1470 (5.2)
Diabetic foot	13374 (12.9)	3409 (11.9)
Osteoporosis	15093 (14.5)	4193 (14.7)
Osteoarthritis	46964 (45.1)	12734 (44.5)
Rheumatoid arthritis	2258 (2.2)	635 (2.2)
Gout	6769 (6.5)	1836 (6.4)
Hypothyroidism	12277 (11.8)	3313 (11.6)
Hyperthyroidism	2557 (2.5)	645 (2.3)
Depression	24945 (23.9)	7505 (26.2)
Anxiety	18780 (18.0)	5485 (19.2)
Chronic liver disease	431 (0.4)	121 (0.4)
**Metabolic characteristics/biomarkers**		
Systolic BP (mmHg)		
<140	63986 (61.4)	19154 (67.0)
≥140	39535 (37.9)	9270 (32.4)
Missing	716 (0.7)	185 (0.6)
Diastolic BP (mmHg)		
<90	97043 (93.1)	26886 (94.0)
≥90	6463 (6.2)	1532 (5.4)
Missing	731 (0.7)	191 (0.7)
Total cholesterol (mmol/L)		
<5.2	52838 (50.7)	15255 (53.3)
5.2 to 6.1	20629 (19.8)	5094 (17.8)
≥6.1	16003 (15.4)	3703 (12.9)
Missing	14767 (14.2)	4557 (15.9)
HDL-cholesterol (mmol/L)		
<1.53	43640 (41.9)	12571 (43.9)
≥1.53	40941 (39.3)	9718 (34.0)
Missing	19656 (18.9)	6320 (22.1)
LDL-cholesterol (mmol/L)		
<2.6	30224 (29.0)	8256 (28.9)
2.6 to 3.4	17672 (17.0)	4573 (16.0)
3.4 to 4.2	11578 (11.1)	2826 (9.9)
4.2 to 4.9	4795 (4.6)	1099 (3.8)
≥4.9	2321 (2.2)	555 (1.9)
Missing	37647 (36.1)	11300 (39.5)
Triglycerides (mmol/L)		

© 2024 Wang J et al. *JAMA Network Open.*

<1.69	60590 (58.1)	15831 (55.3)
1.69 to 2.26	11278 (10.8)	2899 (10.1)
2.26 to 5.63	7464 (7.2)	1888 (6.6)
≥5.63	145 (0.1)	37 (0.1)
Missing	24760 (23.8)	7954 (27.8)
HbA1c (%)		
<5.7	10305 (9.9)	2570 (9.0)
5.7 to 6.5	11478 (11.0)	2614 (9.1)
6.5 to 7.5	4705 (4.5)	1215 (4.2)
≥7.5	3240 (3.1)	893 (3.1)
Missing	74509 (71.5)	21317 (74.5)

Abbreviations: Donep, Donepezil; Riva, Rivastigmine; Gala, Galantamine; SD, standard deviation; BMI, body mass index; IQR, InterQuartile range; BP, blood pressure; HbA1c, glycated haemoglobin A1c; HDL, high-density lipoprotein; LDL, low-density lipoprotein; TIA, transient ischemic attack.

X value to Y value refers to [X, Y)

© 2024 Wang J et al. *JAMA Network Open.*

eTable 5. Baseline Sociodemographic Characteristics, Behavioural/Lifestyle Risk Factors, Dementia-Related Characteristics, Comorbidities, and Metabolic Characteristics/Biomarkers of Unweighted Cohort Two

	Unweighted Cohort Two	
	**Memantine (n = 58,344)**	**Donepezil/ Rivastigmine/ Galantamine (n = 101,075)**
**Sociodemographic characteristics**		
Mean age, years (SD)	81.4 (7.8)	81.1 (7.4)
Male sex, n (%)	24037 (41.2)	40145 (39.7)
Ethnicity, n (%)		
White	34616 (59.3)	61520 (60.9)
South Asian	934 (1.6)	1801 (1.8)
Black	1193 (2.0)	2250 (2.2)
Mixed Race	101 (0.2)	220 (0.2)
Others	292 (0.5)	540 (0.5)
Missing	21208 (36.3)	34744 (34.4)
**Behavioural/lifestyle risk factors**		
Mean BMI kg/m^2 (SD)	25.6 (5.0)	25.66 (4.9)
Smoking status, n (%)		
Non-smokers	21743 (37.3)	38405 (38.0)
Ex-smokers	27034 (46.3)	45866 (45.4)
Current smokers	8765 (15.0)	15191 (15.0)
Missing	802 (1.4)	1613 (1.6)
Drinking status, n (%)		
Non-drinkers	6357 (10.9)	9607 (9.5)
Ex-drinkers	3890 (6.7)	5973 (5.9)
Current drinkers	43437 (74.4)	77680 (76.9)
Missing	4660 (8.0)	7815 (7.7)
**Dementia-related characteristics**		
Median dementia duration in years [IQR]	1.0 [0.3, 2.8]	0.6 [0.2, 2.2]
Vascular dementia, n (%)	8051 (13.8)	8357 (8.3)
Other dementia, n (%)	44208 (75.8)	72495 (71.7)
Alzheimer's disease, n (%)	38430 (65.9)	70089 (69.3)
**Comorbidities**, n (%)		
Hypertension	33059 (56.7)	56205 (55.6)
Chronic kidney disease	15290 (26.2)	25205 (24.9)
Ischemic heart disease	13379 (22.9)	18621 (18.4)
Stroke/TIA	9154 (15.7)	13383 (13.2)
Myocardial infarction	4988 (8.5)	6366 (6.3)

© 2024 Wang J et al. *JAMA Network Open.*

Heart failure	4790 (8.2)	5927 (5.9)
Atrial fibrillation	9144 (15.7)	11471 (11.3)
Peripheral vascular disease	1703 (2.9)	2552 (2.5)
Aortic atheroma & plaque	2696 (4.6)	3902 (3.9)
Type 1 diabetes	402 (0.7)	640 (0.6)
Type 2 diabetes	10009 (17.2)	16988 (16.8)
Peripheral Neuropathy	4787 (8.2)	8371 (8.3)
Diabetic retinopathy	3607 (6.2)	2366 (6.1)
Diabetic foot	9167 (15.7)	15554 (15.4)
Osteoporosis	9616 (16.5)	16392 (16.2)
Osteoarthritis	27244 (46.7)	46900 (46.4)
Rheumatoid arthritis	1300 (2.2)	2282 (2.3)
Gout	4366 (7.5)	7069 (7.0)
Hypothyroidism	7293 (12.5)	12218 (12.1)
Hyperthyroidism	1498 (2.6)	2418 (2.4)
Depression	15572 (26.7)	25103 (24.8)
Anxiety	12053 (20.7)	19108 (18.9)
Chronic liver disease	267 (0.5)	483 (0.5)
**Metabolic characteristics/biomarkers**		
Systolic BP (mmHg)		
<140	40375 (69.2)	67702 (67.0)
≥140	17710 (30.4)	32805 (32.5)
Missing	259 (0.4)	568 (0.6)
Diastolic BP (mmHg)		
<90	55077 (94.4)	95409 (94.4)
≥90	3003 (5.1)	5082 (5.0)
Missing	264 (0.5)	584 (0.6)
Total cholesterol (mmol/L)		
<5.2	33920 (58.1)	55190 (54.6)
5.2 to 6.1	10348 (17.7)	18967 (18.8)
≥6.1	7310 (12.5)	13708 (13.6)
Missing	6766 (11.6)	13210 (13.1)
HDL-cholesterol (mmol/L)		
<1.53	28369 (48.6)	46143 (45.7)
≥1.53	22134 (37.9)	39694 (39.3)
Missing	7841 (13.4)	15238 (15.1)
LDL-cholesterol (mmol/L)		
<2.6	21291 (36.5)	32296 (32.0)
2.6 to 3.4	10195 (17.5)	17177 (17.0)
3.4 to 4.2	6277 (10.8)	10888 (10.8)
4.2 to 4.9	2354 (4.0)	4265 (4.2)
≥4.9	1159 (2.0)	2000 (2.0)
Missing	17068 (29.3)	34449 (34.1)

© 2024 Wang J et al. *JAMA Network Open.*

Triglycerides (mmol/L)		
<1.69	35962 (61.6)	60205 (59.6)
1.69 to 2.26	6976 (12.0)	11643 (11.5)
2.26 to 5.63	4503 (7.7)	7788 (7.7)
≥5.63	89 (0.2)	150 (0.1)
Missing	10814 (18.5)	21289 (21.1)
HbA1c (%)		
<5.7	6705 (11.5)	11204 (11.1)
5.7 to 6.5	7668 (13.1)	12344 (12.2)
6.5 to 7.5	2782 (4.8)	4658 (4.6)
≥7.5	2027 (3.5)	3283 (3.2)
Missing	39162 (67.1)	69586 (68.8)

Abbreviations: Donep, Donepezil; Riva, Rivastigmine; Gala, Galantamine; SD, standard deviation; BMI, body mass index; IQR, InterQuartile range; BP, blood pressure; HbA1c, glycated haemoglobin A1c; HDL, high-density lipoprotein; LDL, low-density lipoprotein; TIA, transient ischemic attack.
X value to Y value refers to [X, Y)

© 2024 Wang J et al. *JAMA Network Open.*

eTable 6. Baseline Sociodemographic Characteristics, Behavioural/Lifestyle Risk Factors, Dementia-Related Characteristics, Comorbidities, and Metabolic Characteristics /Biomarkers of Propensity Score Matched Cohorts (Sensitivity Analyses)

	Matched Cohort One		Matched Cohort Two		Matched Cohort Three	
	**Donepezil**	**Riva|Gala**	**Memantine**	**Done|Riva|Gala**	**Memantine**	**Riva|Gala**
**n**	26808	26808	23890	23890	6482	6482
**Sociodemographic characteristics**						
**Mean age, years (SD)**	80.63 (7.69)	80.70 (7.44)	80.04 (8.10)	79.90 (7.73)	79.51 (7.86)	79.70 (7.67)
**Male sex, n (%)**	10709 (39.9)	11181 (41.7)	9563 (40.0)	9609 (40.2)	2712 (41.8)	2654 (40.9)
**Ethnicity, n (%)**						
White	13099 (48.9)	13477 (50.3)	13845 (58.0)	13987 (58.5)	3592 (55.4)	3576 (55.2)
South Asian	416 (1.6)	334 (1.2)	346 (1.4)	340 (1.4)	80 (1.2)	90 (1.4)
Black	717 (2.7)	479 (1.8)	496 (2.1)	438 (1.8)	100 (1.5)	103 (1.6)
Mixed Race	71 (0.3)	37 (0.1)	44 (0.2)	44 (0.2)	11 (0.2)	15 (0.2)
Others	140 (0.5)	147 (0.5)	121 (0.5)	111 (0.5)	27 (0.4)	31 (0.5)
Missing	12365 (46.1)	12334 (46.0)	9038 (37.8)	8970 (37.5)	2672 (41.2)	2667 (41.1)
**Behavioural/lifestyle risk factors**						
**Mean BMI kg/m^2 (SD)**	25.41 (4.85)	25.31 (4.96)	25.36 (4.53)	25.37 (4.62)	25.24 (4.50)	25.15 (4.72)
**Smoking status, n (%)**						
Non-smokers	10573 (39.4)	10609 (39.6)	9426 (39.5)	9291 (38.9)	2612 (40.3)	2643 (40.8)
Ex-smokers	11382 (42.5)	11535 (43.0)	10746 (45.0)	10771 (45.1)	2896 (44.7)	2850 (44.0)
Current smokers	4189 (15.6)	4062 (15.2)	3374 (14.1)	3471 (14.5)	875 (13.5)	839 (12.9)
Missing	664 (2.5)	602 (2.2)	344 (1.4)	357 (1.5)	99 (1.5)	150 (2.3)
**Drinking status, n (%)**						
Non-drinkers	3129 (11.7)	3158 (11.8)	2563 (10.7)	2619 (11.0)	737 (11.4)	756 (11.7)
Ex-drinkers	1549 (5.8)	1492 (5.6)	1533 (6.4)	1490 (6.2)	383 (5.9)	384 (5.9)

© 2024 Wang J et al. *JAMA Network Open.*

Current drinkers	19894 (74.2)	19975 (74.5)	17857 (74.7)	17731 (74.2)	4794 (74.0)	4628 (71.4)
Missing	2236 (8.3)	2183 (8.1)	1937 (8.1)	2050 (8.6)	568 (8.8)	714 (11.0)
**Dementia-related characteristics**						
**Median dementia duration in years [IQR]**	1.47 (2.19)	1.66 (1.49)	2.38 [1.13, 4.15]	2.18 [0.68, 4.18]	2.40 [1.19, 4.29]	2.35 [0.76, 4.53]
**Vascular dementia, n (%)**	2839 (10.6)	2793 (10.4)	2068 (8.7)	2322 (9.7)	629 (9.7)	706 (10.9)
**Other dementia, n (%)**	21838 (81.5)	22274 (83.1)	21185 (88.7)	21241 (88.9)	5791 (89.3)	5740 (88.6)
**Alzheimer's disease, n (%)**	14988 (55.9)	14477 (54.0)	17615 (73.7)	17294 (72.4)	4233 (65.3)	4192 (64.7)
**Comorbidities, n (%)**						
**Hypertension**	14179 (52.9)	14097 (52.6)	12402 (51.9)	12404 (51.9)	3263 (50.3)	3260 (50.3)
**Chronic kidney disease**	6207 (23.2)	6326 (23.6)	5425 (22.7)	5418 (22.7)	1482 (22.9)	1434 (22.1)
**Ischemic heart disease**	5486 (20.5)	5507 (20.5)	4425 (18.5)	4609 (19.3)	1335 (20.6)	1325 (20.4)
**Stroke/TIA**	3708 (13.8)	3805 (14.2)	3037 (12.7)	3183 (13.3)	847 (13.1)	878 (13.5)
**Myocardial infarction**	1986 (7.4)	2019 (7.5)	1526 (6.4)	1647 (6.9)	462 (7.1)	432 (6.7)
**Heart failure**	1713 (6.4)	1732 (6.5)	1299 (5.4)	1356 (5.7)	347 (5.4)	345 (5.3)
**Atrial fibrillation**	3089 (11.5)	3147 (11.7)	2458 (10.3)	2650 (11.1)	708 (10.9)	736 (11.4)
**Peripheral vascular disease**	735 (2.7)	733 (2.7)	546 (2.3)	562 (2.4)	151 (2.3)	141 (2.2)
**Aortic atheroma & plaque**	1073 (4.0)	1108 (4.1)	907 (3.8)	993 (4.2)	287 (4.4)	278 (4.3)
**Type 1 diabetes**	170 (0.6)	168 (0.6)	137 (0.6)	152 (0.6)	41 (0.6)	50 (0.8)
**Type 2 diabetes**	4175 (15.6)	4048 (15.1)	3494 (14.6)	3592 (15.0)	910 (14.0)	920 (14.2)
**Peripheral Neuropathy**	1915 (7.1)	1869 (7.0)	1747 (7.3)	1743 (7.3)	429 (6.6)	486 (7.5)
**Diabetic retinopathy**						
No Diabetic retinopathy	25401 (94.8)	25421 (94.8)	22676 (94.9)	22600 (94.6)	6154 (94.9)	6142 (94.8)
Background Diabetic retinopathy	942 (3.5)	940 (3.5)	833 (3.5)	878 (3.7)	233 (3.6)	229 (3.5)
STDR	465 (1.7)	447 (1.7)	381 (1.6)	412 (1.7)	95 (1.5)	111 (1.7)
**Diabetic foot**						
No diabetic foot	23598 (88.0)	23686 (88.4)	20833 (87.2)	20757 (86.9)	5747 (88.7)	5752 (88.7)
Diabetic foot	2878 (10.7)	2811 (10.5)	2833 (11.9)	2896 (12.1)	679 (10.5)	672 (10.4)

© 2024 Wang J et al. *JAMA Network Open.*

Diabetic foot ulcer	332 (1.2)	311 (1.2)	224 (0.9)	237 (1.0)	56 (0.9)	58 (0.9)
**Osteoporosis**	3782 (14.1)	3943 (14.7)	3772 (15.8)	3723 (15.6)	1067 (16.5)	1071 (16.5)
**Osteoarthritis**	11876 (44.3)	12017 (44.8)	10723 (44.9)	10700 (44.8)	2938 (45.3)	2870 (44.3)
**Rheumatoid arthritis**	631 (2.4)	603 (2.2)	465 (1.9)	488 (2.0)	140 (2.2)	136 (2.1)
**Gout**	1723 (6.4)	1718 (6.4)	1562 (6.5)	1513 (6.3)	395 (6.1)	377 (5.8)
**Hypothyroidism**	3163 (11.8)	3149 (11.7)	2834 (11.9)	2869 (12.0)	802 (12.4)	805 (12.4)
**Hyperthyroidism**	635 (2.4)	619 (2.3)	599 (2.5)	565 (2.4)	157 (2.4)	152 (2.3)
**Depression**	6825 (25.5)	6960 (26.0)	6502 (27.2)	6715 (28.1)	1839 (28.4)	1852 (28.6)
**Anxiety**	4921 (18.4)	5043 (18.8)	5110 (21.4)	5175 (21.7)	1468 (22.6)	1424 (22.0)
**Chronic liver disease**	113 (0.4)	118 (0.4)	87 (0.4)	97 (0.4)	23 (0.4)	30 (0.5)
**Metabolic characteristics/biomarkers**						
**Systolic BP (mmHg)**						
<140	17062 (63.6)	17695 (66.0)	16917 (70.8)	16870 (70.6)	4637 (71.5)	4596 (70.9)
≥140	9527 (35.5)	8938 (33.3)	6877 (28.8)	6897 (28.9)	1816 (28.0)	1836 (28.3)
Missing	219 (0.8)	175 (0.7)	96 (0.4)	123 (0.5)	29 (0.4)	50 (0.8)
**Diastolic BP (mmHg)**						
<90	24983 (93.2)	25159 (93.8)	22617 (94.7)	22604 (94.6)	6149 (94.9)	6122 (94.4)
≥90	1604 (6.0)	1469 (5.5)	1174 (4.9)	1157 (4.8)	302 (4.7)	309 (4.8)
Missing	221 (0.8)	180 (0.7)	99 (0.4)	129 (0.5)	31 (0.5)	51 (0.8)
**Total cholesterol (mmol/L)**						
<5.2	13998 (52.2)	14139 (52.7)	12998 (54.4)	13055 (54.6)	3512 (54.2)	3357 (51.8)
5.2 to 6.1	4907 (18.3)	4825 (18.0)	4557 (19.1)	4318 (18.1)	1220 (18.8)	1176 (18.1)
≥6.1	3630 (13.5)	3589 (13.4)	3402 (14.2)	3317 (13.9)	878 (13.5)	858 (13.2)
Missing	4273 (15.9)	4255 (15.9)	2933 (12.3)	3200 (13.4)	872 (13.5)	1091 (16.8)
**HDL-cholesterol (mmol/L)**						
<1.53	11493 (42.9)	11588 (43.2)	10987 (46.0)	11104 (46.5)	3002 (46.3)	2860 (44.1)
≥1.53	9435 (35.2)	9305 (34.7)	9412 (39.4)	9188 (38.5)	2411 (37.2)	2372 (36.6)
Missing	5880 (21.9)	5915 (22.1)	3491 (14.6)	3598 (15.1)	1069 (16.5)	1250 (19.3)

© 2024 Wang J et al. *JAMA Network Open.*

**LDL-cholesterol (mmol/L)**						
<2.6	7902 (29.5)	7648 (28.5)	7851 (32.9)	8019 (33.6)	2065 (31.9)	1994 (30.8)
2.6 to 3.4	4233 (15.8)	4335 (16.2)	4185 (17.5)	4102 (17.2)	1089 (16.8)	1071 (16.5)
3.4 to 4.2	2690 (10.0)	2686 (10.0)	2786 (11.7)	2675 (11.2)	728 (11.2)	693 (10.7)
4.2 to 4.9	1063 (4.0)	1057 (3.9)	1062 (4.4)	1024 (4.3)	287 (4.4)	270 (4.2)
≥4.9	556 (2.1)	539 (2.0)	548 (2.3)	555 (2.3)	152 (2.3)	134 (2.1)
Missing	10364 (38.7)	10543 (39.3)	7458 (31.2)	7515 (31.5)	2161 (33.3)	2320 (35.8)
**Triglycerides (mmol/L)**						
<1.69	15064 (56.2)	14784 (55.1)	14402 (60.3)	14333 (60.0)	3771 (58.2)	3637 (56.1)
1.69 to 2.26	2787 (10.4)	2738 (10.2)	2876 (12.0)	2892 (12.1)	765 (11.8)	731 (11.3)
2.26 to 5.63	1875 (7.0)	1794 (6.7)	1806 (7.6)	1774 (7.4)	455 (7.0)	427 (6.6)
≥5.63	36 (0.1)	36 (0.1)	35 (0.1)	34 (0.1)	9 (0.1)	6 (0.1)
Missing	7046 (26.3)	7456 (27.8)	4771 (20.0)	4857 (20.3)	1482 (22.9)	1681 (25.9)
**eGFR (%)**						
<30	294 (1.1)	362 (1.4)	215 (0.9)	215 (0.9)	81 (1.2)	71 (1.1)
30 to 60	2729 (10.2)	2472 (9.2)	2460 (10.3)	2407 (10.1)	658 (10.2)	671 (10.4)
60 to 90	4373 (16.3)	4413 (16.5)	4413 (18.5)	4356 (18.2)	1215 (18.7)	1181 (18.2)
⩾90	524 (2.0)	469 (1.7)	445 (1.9)	452 (1.9)	99 (1.5)	100 (1.5)
Missing	18888 (70.5)	19092 (71.2)	16357 (68.5)	16460 (68.9)	4429 (68.3)	4459 (68.8)
**HbA1c (%)**						
<5.7	2535 (9.5)	2413 (9.0)	2804 (11.7)	2684 (11.2)	686 (10.6)	660 (10.2)
5.7 to 6.5	2918 (10.9)	2469 (9.2)	2992 (12.5)	2957 (12.4)	746 (11.5)	691 (10.7)
6.5 to 7.5	1248 (4.7)	1152 (4.3)	981 (4.1)	992 (4.2)	275 (4.2)	282 (4.4)
≥7.5	856 (3.2)	862 (3.2)	683 (2.9)	727 (3.0)	192 (3.0)	180 (2.8)
Missing	19251 (71.8)	19912 (74.3)	16430 (68.8)	16530 (69.2)	4583 (70.7)	4669 (72.0)

Abbreviations: Donep, Donepezil; Riva, Rivastigmine; Gala, Galantamine; SD, standard deviation; BMI, body mass index; IQR, InterQuartile range; BP, blood pressure; HbA1c, glycated haemoglobin A1c; HDL, high-density lipoprotein; LDL, low-density lipoprotein; TIA, transient ischemic attack; STDR, sight-threatening diabetic retinopathy.

© 2024 Wang J et al. *JAMA Network Open.*

Data were presented for the first multiple imputed propensity-score matched cohort.
X value to Y value refers to [X, Y).

© 2024 Wang J et al. *JAMA Network Open.*

eTable 7. Risk of Developing Age-Related Macular Degeneration in Donepezil/Memantine Users and Comparator Drug Users in CPRD GOLD and CPRD Aurum

	Crude HR (95% CI)	Adjusted HR* (95% CI)
**Cohort One†**	**1.01 (0.72, 1.44)**	**0.95 (0.67, 1.35)**
**GOLD**	0.84 (0.71, 1.00)	0.67 (0.55, 0.81)
**Aurum**	1.21 (1.08, 1.34)	1.11 (0.99, 1.25)
**Cohort Two†**	**1.20 (0.99, 1.44)**	**1.03 (0.83, 1.27)**
**GOLD**	1.17 (0.77, 1.77)	1.23 (0.78, 1.94)
**Aurum**	1.21 (0.99, 1.48)	0.98 (0.77, 1.24)
**Cohort Three†**	**1.19 (0.87, 1.63)**	**1.24 (0.83, 1.86)**
**GOLD**	0.94 (0.50, 1.79)	1.38 (0.65, 2.95)
**Aurum**	1.28 (0.89, 1.85)	1.19 (0.74, 1.92)

Abbreviations: HRs, Hazard ratios;
* Standardized mortality ratio weighted analyses adjusted for age, sex, BMI categories, ethnicity, index year, data source, smoking status, drinking status, dementia duration, vascular dementia, other dementia, Alzheimer's, Memantine use (Cohort One only), preceding comparator drug prescription counts (Cohort Two/Three only), hypertension, chronic kidney disease, ischemic heart disease, stroke or transient ischemic attack, myocardial infarction, heart failure, atrial fibrillation, peripheral vascular disease, aortic atheroma and plaque, type 1 diabetes, type 2 diabetes, peripheral neuropathy, diabetic retinopathy, diabetic foot, osteoporosis, osteoarthritis, rheumatoid arthritis, gout, hypothyroidism, hyperthyroidism, depression, anxiety, chronic liver disease, systolic blood pressure, diastolic blood pressure, total cholesterol, high-density lipoprotein cholesterol, low-density lipoprotein cholesterol, triglycerides, and glycated haemoglobin A1c.
† Analyses were performed separately for GOLD and Aurum and pooled using IPD meta-analyses.

© 2024 Wang J et al. *JAMA Network Open.*
