# Supplementary material for: Pharmacologic Treatments for Dementia and the Risk of Developing Age-Related Macular Degeneration
Source: JAMA Netw Open. 2024 Oct 24;7(10):e2441166. doi: 10.1001/jamanetworkopen.2024.41166 (PMC11581610; doi:10.1001/jamanetworkopen.2024.41166)
Supplement: Supplement 2. — Data Sharing Statement [file jamanetwopen-e2441166-s002.pdf]

Data Sharing Statement

Wang. Pharmacologic Treatments for Dementia and the Risk of Developing Age-Related Macular Degeneration. *JAMA Netw Open*. Published October 24, 2024.
doi:10.1001/jamanetworkopen.2024.41166

Data
Data available: No

Additional Information
Explanation for why data not available: The data that support the findings of this study are available from CPRD but restrictions apply to the availability of these data, which were used under license for the current study, and so are not publicly available. Data are available from CPRD following submission of a protocol and approval by CPRD Research Data Governance.
